# The SIRT1–p53 axis drives a ferro-aging-like program and aggravates trophoblast dysfunction in preeclampsia

**DOI:** 10.3389/fragi.2026.1838730

**Published:** 2026-06-03

**Authors:** Rong Hu, Zhi Chen, Wei Bian, Jing Zhang, Xiaoqin Xu, Hui Xiong, Shaojian Xiang, Yubin Ding, Hongbo Qi, Huan Yang

**Affiliations:** 1 Department of Obstetrics, Chongqing University Three Gorges Hospital, Chongqing, China; 2 Department of Gynecology, Chongqing Traditional Chinese Medicine Hospital, Chongqing, China; 3 Department of Teaching, Chongqing University Three Gorges Hospital, Chongqing, China; 4 Department of Obstetrics and Gynecology, Women and Children’s Hospital of Chongqing Medical University, Chongqing, China; 5 Department of Pharmacology, Academician Workstation, School of Pharmacology, Changsha Medical University, Changsha, Hunan, China; 6 Department of Obstetrics, The First Affiliated Hospital of Chongqing Medical University, Chongqing, China

**Keywords:** ferro-aging-like, iron dyshomeostasis, preeclampsia, premature placental senescence, SIRT1, trophoblast dysfunction

## Abstract

Preeclampsia (PE) is a pregnancy-specific syndrome driven by placental dysfunction, and premature placental senescence has increasingly been implicated in its pathogenesis. However, the upstream regulatory mechanisms remain poorly understood. Here, we found that placental SIRT1 expression was reduced in PE and was accompanied by senescence-associated features. In trophoblasts, SIRT1 knockdown enhanced senescence and senescence-associated secretory phenotype (SASP) release, while impairing proliferative capacity, migration, and invasion, whereas pharmacological activation of SIRT1 attenuated placental senescence and ameliorated PE-like manifestations *in vivo*. Mechanistically, iron chelation alleviated senescence, whereas senolytic intervention partially restored iron homeostasis, supporting a self-reinforcing interaction between iron dyshomeostasis and senescence. Collectively, these findings identify the SIRT1–p53 axis as an upstream regulator linking iron dyshomeostasis to placental senescence, support the existence of a ferro-aging-like pathogenic program in diseased placentas, and provide new insights into the molecular basis of placental dysfunction in PE.

## 1 Introduction

Preeclampsia (PE) is a pregnancy-specific disorder characterized by hypertension and often accompanied by proteinuria and multiorgan dysfunction. It affects approximately 2%–5% of pregnancies worldwide and remains a major cause of adverse maternal and perinatal outcomes (Chappell et al., 2021; Dimitriadis et al., 2023). Although the pathogenesis of PE is complex, placental pathology is widely considered as central to disease development. Defective trophoblast function limits invasion and impairs remodeling of the uterine spiral arteries, resulting in inadequate placental perfusion, hypoxia–reoxygenation injury, and subsequent maternal systemic dysfunction (Rana et al., 2019; Staff, 2019; Redman et al., 2022). Clarifying the molecular basis of trophoblast dysfunction is therefore essential for understanding PE and identifying new therapeutic strategies.

Cellular senescence is a stable state of cell-cycle arrest induced by various forms of stress. It is characterized by increased expression of cell-cycle inhibitors such as p16 and p21, together with the development of the senescence-associated secretory phenotype (SASP). Through the release of inflammatory cytokines, chemokines, and matrix-remodeling factors, SASP reshapes the local microenvironment and promotes disease progression (Wang et al., 2024). The placenta is highly metabolically active organ and is particularly sensitive to stress. During normal gestation, it undergoes a degree of physiological senescence-like change. Under pathological conditions, however, premature or excessive senescence may compromise placental function. Previous studies have reported increased senescence markers and inflammatory mediators in PE placentas, suggesting that premature placental senescence contributes to PE-associated placental insufficiency (Cox and Redman, 2017; Scaife et al., 2021; Redman et al., 2022; Roh et al., 2024; Sugulle et al., 2024). However, the upstream molecular program driving these changes remains unclear, as does the extent to which they directly contribute to trophoblast dysfunction.

Sirtuin 1 (SIRT1) is an NAD + -dependent deacetylase that regulates metabolic homeostasis, mitochondrial function, oxidative stress responses, and cell fate decisions (Liu et al., 2022; Yu et al., 2023). In many tissues, SIRT1 exerts anti-senescent and anti-inflammatory effects. In trophoblasts, it also regulates proliferation, migration, and invasion (Liu et al., 2022; Yu et al., 2023). P53 is a well-established substrate of SIRT1. By deacetylating p53, SIRT1 modulates its transcriptional activity and thereby influences cell-cycle arrest, apoptosis, and senescence (Xiong et al., 2021). These observations suggest that the SIRT1–p53 axis may participate in placental stress responses in PE. However, direct evidence that SIRT1 dysregulation acts upstream of trophoblast senescence is still lacking.

Disordered iron metabolism and oxidative stress have recently emerged as important contributors to placental injury in PE (Chen Z. et al., 2022; Yang et al., 2026). Iron is essential for placental and fetal development (Zhang et al., 2022). Excess labile iron, however, amplifies reactive oxygen species (ROS) generation through the Fenton reaction and promotes lipid peroxidation, mitochondrial dysfunction, and cellular injury (Tang et al., 2021). Iron dyshomeostasis may also enhance senescence-associated phenotypes and SASP(Maus et al., 2023; Yang et al., 2026). Emerging evidence further suggests that iron-dependent lipid peroxidation can promote senescence-associated programs beyond classical ferroptosis, giving rise to a ferro-aging-like state in certain pathological settings (Liu et al., 2026). Whether a similar iron-dependent, senescence-promoting process operates in PE placentas, and whether it is linked to SIRT1-related signaling, remains unknown.

Based on these observations, we hypothesized that SIRT1 dysregulation acts as an upstream regulator linking trophoblast senescence, iron dyshomeostasis, and oxidative stress in PE. To test this hypothesis, we combined clinical placental samples with cellular and animal models to determine whether SIRT1-related signaling couples disturbed iron metabolism to the senescence program and to define the underlying molecular mechanisms. This work may provide new insights into pathogenesis of PE and suggest potential therapeutic targets.

## 2 Materials and methods

### 2.1 Study participants and sample collection

This study was conducted in accordance with the Declaration of Helsinki and approved by the Ethics Committee of Chongqing University Three Gorges Hospital (approval No. 2023–182). Written informed consent was obtained from all participants. All participants had singleton pregnancies and underwent cesarean delivery. Cases with gestational diabetes mellitus, chronic kidney disease, premature rupture of membranes, fetal chromosomal abnormalities, or other major complications were excluded. PE was diagnosed according to the American College of Obstetricians and Gynecologists (ACOG) Practice Bulletin No. 222 (Author anonymous, 2020). Placental tissues were collected immediately after delivery. Tissue blocks (∼1.5 × 1.5 cm) were excised from the central region of the maternal surface, avoiding areas of infarction and calcification. Samples were washed with PBS. Part was fixed in 4% paraformaldehyde for paraffin embedding, and the remainder was snap-frozen in liquid nitrogen and stored at −80 °C. A total of 30 women with normal term pregnancies and 30 women with early-onset PE were included. Detailed clinical characteristics are shown in Supplementary Table S1.

### 2.2 Cell culture

The immortalized human trophoblast cell line HTR-8/SVneo was obtained from the American Type Culture Collection (ATCC, Manassas,VA, United States) and cultured in Roswell Park Memorial Institute (RPMI) 1640 medium (Cat. No.11875093; Gibco, New York, United States) supplemented with 10% Fetal Bovine Serum (FBS) (Cat. No. ST30-2602; PAN-Biotech, Adenbach, Germany) and 1% penicillin-streptomycin (Cat. No. C0222; Beyotime, Shanghai, China). Cells were maintained at 37 °C in a humidified incubator with 5% CO_2_. Erastin (Cat. No. S7242; Selleck, Houston, TX, United States), Deferoxamine-1 (Cat. No. S5742; Selleck, Houston, TX, United States), SRT1720(Cat. No. HY-10532; MedChemExpress, United States) and procyanidin C1 (Cat. No. E0478; Selleck, Houston, TX, United States) were dissolved in dimethyl sulfoxide (DMSO) (Cat. No. D1435; Sigma-Aldrich, United States) to prepare stock solutions.

### 2.3 Animal experiments

Female CD-1 mice aged 8–12 weeks and weighing 25–35 g were obtained from Chongqing Byrness Weil Biotechnology Co., Ltd (Chongqing, China) and mated with age-matched males. All mice were housed in the animal facility of Chongqing University Three Gorges Hospital, under controlled temperature conditions (23 °C) with a 12-h light/12-h dark cycle. The presence of a vaginal plug was designated embryonic day 0.5 (E0.5). Pregnant mice were randomly assigned to the Control, L-NAME, and L-NAME + SRT2104 groups. From E8.5 to E18.5, mice in the L-NAME and L-NAME + SRT2104 groups received L-NAME (100 mg/kg/day; Cat. No. N5751; Sigma-Aldrich, St. Louis, United States) by oral gavage (de Alwis et al., 2022). Mice in the L-NAME + SRT2104 group additionally received intraperitoneal SRT2104 (25 mg/kg/day; Cat. No. HY-15262; MedChemExpress, Monmouth Junction, NJ, United States), as previously described (Pei et al., 2022). Control mice received the corresponding vehicle. Systolic blood pressure (SBP) was measured daily from E6.5 to E18.5 using a BP-2000 Series II tail-cuff system (Visitech Systems, Apex, NC, United States). Mice were euthanized at E18.5, and placental and maternal tissues were collected for further analyses. Animal experiments were reported in accordance with the ARRIVE 2.0 guidelines (Percie du Sert et al., 2020).

### 2.4 Immunohistochemistry

Tissue fixation and sectioning were performed as previously described (Yang et al., 2022). Placental sections were incubated overnight at 4 °C with primary antibodies against p16 (1:1,000; Cat. No. 10883-1-AP; Proteintech, Wuhan, China), p21 (1:1,000; Cat. No. 10355-1-AP; Proteintech, Wuhan, China), SIRT1 (1:500; Cat. No. Ab189494; Abcam, Cambridge, United Kingdom), FTH (1:200; Cat. No. 11682-1-AP; Proteintech, Wuhan, China), TFR (1:200; Cat. No. WL03500; Wanleibio, Shenyang, China), FPN1(1:100; Cat. No. 26601-1-AP; Proteintech, Wuhan, China), CK7 (1:200; Cat. No. 17513-1-AP; Proteintech, Wuhan, China), and HLA-G (1:500; Cat. No. 66447-1-Ig; Proteintech, Wuhan, China). After washing, sections were incubated with the appropriate HRP-conjugated secondary antibodies for 30 min at room temperature. Signals were visualized with 3,3′-diaminobenzidine (DAB), and images were acquired using an EVOS microscope (Life Technologies, United States). Positive staining was quantified using ImageJ 1.50i software (NIH, Bethesda, MD, United States). Three independent experimental replicates were conducted, with three random fields analyzed per sample.

### 2.5 SA-β-gal staining

Senescence-associated β-galactosidase (SA-β-gal) staining was performed using a commercial kit (Cat. No. C0602; Beyotime, Shanghai, China) according to the manufacturer’s instructions. Briefly, cells, villi, and fresh placental tissues were fixed in fixative solution (formaldehyde-glutaraldehyde mix) for 15 min at room temperature and then incubated with staining solution overnight at 37 °C in a CO_2_-free incubator. Staining was visualized by light microscopy, and images were captured (Leica camera). Blue-stained areas were considered SA-β-gal-positive and quantified using ImageJ 1.50i software (NIH, Bethesda, MD, United States). Three random fields were analyzed for each sample.

### 2.6 H&E staining

Placentas and kidneys were sectioned into 3 mm-thick pieces after being fixed in 4% paraformaldehyde. Sections were deparaffinized, rehydrated, stained with hematoxylin for 5 min and eosin for 2 min, and imaged using an EVOS microscope (Life Technologies, Carlsbad, CA, United States).

### 2.7 Perls’ prussian blue staining

Iron deposition in placental sections was evaluated using Perls’ Prussian blue staining, as previously described (Yang et al., 2022). Briefly, paraffin-embedded placental sections were deparaffinized and incubated with Perls’ solution for 30 min, followed by washing with PBS. Endogenous peroxidase activity was quenched with 0.3% hydrogen peroxide in methanol for 15 min. Signals were then developed with DAB for 15 min, followed by hematoxylin counterstaining. Images were acquired using an EVOS microscope (Life Technologies, Carlsbad, CA, United States). Positive staining was quantified using ImageJ 1.50i software (NIH, Bethesda, MD, United States), and three random fields were analyzed for each sample.

### 2.8 Immunofluorescence

Placental sections were fixed in 4% paraformaldehyde for 10 min, washed three times with PBS, permeabilized with PBST containing 0.05% Triton X-100 (Cat. No. P0096; Beyotime, Shanghai, China), and blocked with 5% goat serum. Sections were incubated overnight at 4 °C with a mouse monoclonal antibody against SIRT1 (1:100; ab110304; Abcam, Cambridge, United Kingdom) together with rabbit antibodies against p16 (1:100; ab189034; Abcam, Cambridge, United Kingdom) or p21 (1:500; ab188224; Abcam, Cambridge, United Kingdom). After washing with PBS, sections were incubated with species-specific fluorophore-conjugated secondary antibodies for 1 h at room temperature. Nuclei were counterstained with DAPI. Images were acquired using an EVOS microscope (Life Technologies, Carlsbad, CA, United States). Fluorescence intensity was quantified using ImageJ 1.50i software (NIH, Bethesda, MD, United States). Three random fields were analyzed for each sample.

### 2.9 Western blotting

The protein immunoblotting method was referenced from our previous research protocol (Yang et al., 2022). Primary antibodies against SIRT1 (1:1,000; Cat. No. ab189494; Abcam, Cambridge, United Kingdom), acetyl-p53 (Lys382) (1:2000; Cat. No. ab75754; Abcam, Cambridge, United Kingdom), p16INK4a (1:1,000; Cat. No. ab189034; Abcam, Cambridge, United Kingdom), p21 (1:1,000; Cat. No. ab109199; Abcam, Cambridge, United Kingdom), GPX4 (1:1,000; Cat. No. ab125066; Abcam, Cambridge, United Kingdom), FTH1(1:1,000; Cat. No. ab75973; Abcam, Cambridge, United Kingdom), TFR (1:500; Cat. No. WL03500; Wanleibio, Shenyang, China), p53 (1:5,000; Cat. No. 10442-1-AP; Proteintech, China), FPN1 (1:1,000; Cat. No. 26601-1-AP; Proteintech, China), SLC7A11 (1:2000; Cat. No. 26864-1-AP; Proteintech, China), β-tubulin (1:5,000; Cat. No. 10068-1-AP; Proteintech, China), and GAPDH (1:10,000; Cat. No. 60004-1-Ig; Proteintech, China) were purchased from Proteintech (Shanghai, China).

### 2.10 Coimmunoprecipitation (Co-IP)

Reciprocal co-immunoprecipitation was performed in HTR-8/SVneo cells using anti-SIRT1 antibody (Cat. No. ab189494; Abcam, Cambridge, United Kingdom), anti-p53 antibody (Cat. No. 10442-1-AP; Proteintech, China), or the corresponding isotype-matched IgG control (Cat. No. 5946; Cell Signaling Technology, Danvers, MA, United States). Antibodies were pre-incubated with Protein A/G magnetic beads (Cat. No. B23202; Bimake, Houston, TX, United States) for 1.5 h at room temperature. Cells were lysed in IP lysis buffer (Cat. No. 87787; Thermo Fisher Scientific, Waltham, MA, United States). Equal amounts of protein lysate (400 μg per sample) were then incubated with the antibody–bead complexes for 8 h at 4 °C with gentle rotation. A fraction of each lysate was reserved as input control. Immune complexes were collected using a magnetic rack and washed three times with cold IP lysis buffer. Bound proteins were eluted by boiling the bead complexes in diluted 4× Laemmli sample buffer (Cat. No. 1610747; Bio-Rad, Hercules, CA, United States) at 95 °C for 5 min. The immunoprecipitated proteins were subsequently separated by SDS-PAGE and subjected to immunoblotting with the indicated antibodies.

### 2.11 Cell Counting Kit-8 assay

Cell viability was assessed using a Cell Counting Kit-8 (CCK-8; Cat. No. HY-K0301; MedChemExpress, Monmouth Junction, NJ, United States) according to the manufacturer’s instructions. Briefly, HTR-8/SVneo cells were seeded in 96-well plates at a density of 5 × 10^3^ cells per well in 100 μL complete medium. After the indicated treatments, 10 μL of CCK-8 reagent was added to each well, and the cells were incubated for 4 h at 37 °C. Absorbance was then measured at 450 nm using a microplate reader (Thermo Fisher Scientific, United States).

### 2.12 ELISA

The levels of IL-6 (Cat. No. PI330), IL-1α (Cat. No. PI565), and IL-1β (Cat. No. PI305) in culture supernatants, as well as IL-6 (Cat. No. PI326), IL-1α (Cat. No. PI561), and IL-1β (Cat. No. PI301) in mouse placental tissue homogenates, were quantified using ELISA kits (Beyotime Biotechnology, Shanghai, China) according to the manufacturers’ instructions. Absorbance at 450 nm was measured using a Multiskan FC microplate reader (Thermo Scientific, United States), and cytokine concentrations were calculated from standard curves.

### 2.13 Assay for GSH, MDA and iron

Kits were used to measure the levels of GSH (Cat. A006-1–1; Nanjing Jiancheng Bioengineering Institute, Nanjing, China), MDA (Cat. S0131S; Beyotime Biotechnology, Shanghai, China), and iron (Cat. E1042-100; Applygen Technology, Beijing, China) in placental tissues and cells according to the manufacturer’s instructions. Briefly, after the lysis of tissue samples or cells, the supernatant was collected after centrifugation at 10,000–12,000×g for 10 min at 4 °C The content was measured on a colorimetric microplate reader (Thermo Scientific, Multiskan, FC, United States). Protein concentrations were measured using a protein assay kit (Cat. No. P0010; Beyotime Biotechnology, Shanghai, China), and all values were normalized to protein concentration.

### 2.14 Determination of reactive oxygen species

Intracellular ROS levels were measured using the fluorescent probe 2′,7′-dichlorodihydrofluorescein diacetate (DCFH-DA; Cat. No. S0033S; Beyotime, Shanghai, China) according to the manufacturer’s instructions. After the indicated treatments, cells were incubated with DCFH-DA working solution in serum-free medium at 37 °C in the dark for 30 min. Cells were then washed twice with PBS to remove excess probe, harvested by trypsinization, and collected for flow cytometric analysis using a CytoFLEX flow cytometer (Beckman Coulter, United States). A minimum of 10,000 cells was analyzed for each sample.

### 2.15 Assessment of lipid peroxidation

Lipid peroxidation was assessed using the C11-BODIPY™ 581/591 probe (Cat. No. L267; Dojindo, Japan). After the indicated treatments, cells were incubated with 20 μM C11-BODIPY 581/591 in the dark for 1 h, washed twice with PBS to remove excess probe, and maintained in serum-free medium during imaging. Fluorescence images were acquired using an EVOS microscope (Life Technologies, United States). Lipid peroxidation was quantified using ImageJ 1.50i software by analyzing the oxidized (green) and reduced (red) fluorescence signals. Three random fields were analyzed for each sample in three independent experiments.

### 2.16 Cellular labile iron detection

Cellular labile Fe^2+^ levels were assessed using the FerroOrange probe (Cat. No. F374; Dojindo, Japan) according to the manufacturer’s instructions (Yang et al., 2022). After the indicated treatments, cells were incubated with 1 μM FerroOrange in the dark for 30 min at 37 °C. Cells were then washed twice with PBS, and fluorescence images were acquired using an EVOS microscope (Life Technologies, United States). FerroOrange fluorescence intensity was quantified using ImageJ 1.50i software. Three random fields were analyzed for each sample in three independent experiments.

### 2.17 Mitochondrial membrane potential analysis

Mitochondrial membrane potential (ΔΨm) was assessed using the JC-1 dye (Cat. No. C2003S; Beyotime, Shanghai, China) according to the manufacturer’s instructions. After the indicated treatments, cells were incubated with JC-1 working solution (1×) for 30 min at 37 °C, washed twice with PBS, and mounted with anti-fade reagent. Fluorescence images were acquired using a fluorescence microscope. Relative ΔΨm was evaluated by the ratio of red fluorescence (JC-1 aggregates) to green fluorescence (JC-1 monomers) (Sivandzade et al., 2019). The fluorescence ratio was quantified using ImageJ 1.50i software. Three random fields were analyzed for each sample in three independent experiments.

### 2.18 Transmission electron microscopy

Cells were fixed in 3% glutaraldehyde, followed by postfixation in 1% osmium tetroxide. Samples were dehydrated through a graded ethanol series, embedded in Epon 812 resin, and cured at 60 °C for 24 h. Ultrathin sections (50 nm) were cut, mounted on 200-mesh copper grids, and double-stained with uranyl acetate and lead citrate. Images were acquired using a JEM-1400FLASH transmission electron microscope (JEOL, Tokyo, Japan).

### 2.19 Matrigel invasion assay

HTR-8/SVneo cells (5 × 10^4^ cells/well) were resuspended in serum-free RPMI-1640 medium and seeded into the upper chambers of 24-well Transwell inserts (8μm; BD Falcon, United States) pre-coated with diluted Matrigel (Corning, United States). After 24 h, non-invaded cells were removed from the upper surface of the membrane, and invaded cells on the lower surface were fixed, stained with crystal violet, and imaged using an EVOS microscope (Life Technologies, United States). Cell invasion was quantified using ImageJ 1.50i software.

### 2.20 Wound healing assay

HTR-8/SVneo cells were plated in six-well plates and grown to more than 90% confluence. A linear wound was created using a 100 μL pipette tip. After washing with PBS to remove detached cells, fresh medium was added, and images were acquired at 0 and 12 h. Wound closure was quantified using ImageJ 1.50i software by measuring the wound area.

### 2.21 Cell proliferation (EdU staining)

HTR-8/SVneo cells were seeded in 96-well plates at a density of 5 × 10^3^ cells per well. After the indicated treatments, cells were incubated with 100 μL medium containing 50 μM EdU for 2 h, fixed with 4% paraformaldehyde for 30 min, and stained using an EdU detection kit (Cat. No. C10310-1; RiboBio, Guangzhou, China) according to the manufacturer’s instructions. Nuclei were counterstained with Hoechst for 30 min at room temperature. Images were acquired using an EVOS microscope (Life Technologies, United States). Cell proliferation was evaluated by calculating the percentage of EdU-positive cells using ImageJ 1.50i software. Three random fields were analyzed for each sample in three independent experiments.

### 2.22 Transfection

Lentiviral vectors carrying short hairpin RNA (shRNA) targeting human SIRT1 (GV248) and a negative control shRNA (sh-NC) were purchased from GeneChem (Shanghai, China). HTR-8/SVneo cells (4 × 10^5^) were transduced with lentivirus at a multiplicity of infection (MOI) of 10 in the presence of HiTransG A infection enhancer (GeneChem, Shanghai, China) according to the manufacturer’s instructions. Forty-eight hours after transduction, puromycin (0.5 μg/mL) was added for selection, and stable cell lines were established after 10 days of screening.

Small interfering RNA targeting p53 (si-p53) and a negative control siRNA (si-NC) were synthesized by Tsingke Biotechnology (Beijing, China). HTR-8/SVneo cells at 60%–70% confluence was transfected with 100 nM siRNA in six-well plates using Lipofectamine 2000 (Thermo Fisher Scientific, Waltham, MA, United States) according to the manufacturer’s instructions. The shRNA and siRNA sequences used in this study are listed in Supplementary Tables S2, S3.

### 2.23 mRNA sequencing

HTR-8/SVneo cells stably expressing sh-SIRT1 or sh-NC were cultured to 60%–70% confluence. Total RNA was extracted using TRIzol reagent (Cat. No. 15596018; Invitrogen, Carlsbad, CA, United States). RNA quantity and integrity were assessed using an Agilent 2,100 Bioanalyzer (Agilent Technologies, Santa Clara, CA, United States). A total of 1 μg RNA per sample was used for library preparation with the NEBNext® Ultra Directional RNA Library Prep Kit for Illumina (NEB, United States). Libraries were sequenced on an Illumina platform to generate 150-bp reads. Three biological replicates were included in each group (n = 3). Differential expression analysis was performed using the DESeq2 package in R (version 4.2.1). Differentially expressed genes (DEGs) were identified using the criteria of P < 0.01 and |log2 fold change| > 1. Gene Ontology (GO) and Kyoto Encyclopedia of Genes and Genomes (KEGG) enrichment analyses were performed using the clusterProfiler package (version 4.4.4).

### 2.24 Bioinformatics analysis

A search of the Gene Expression Omnibus (GEO) database was performed using the keywords “placenta” and “SIRT1” to identify publicly available placental transcriptomic datasets related to SIRT1. GSE100279 was the only dataset that met the predefined inclusion criteria and was therefore selected for analysis (Arul Nambi Rajan et al., 2018). Differential expression analysis was performed using the limma package in R (version 4.2.1). Genes with P < 0.05 and |log2 fold change| > 1 were defined as DEGs. GO and KEGG enrichment analyses were subsequently performed using the clusterProfiler package (version 4.4.4). This dataset was used to support SIRT1-associated pathway alterations in placenta.

### 2.25 Statistical analyses

Data are presented as mean ± SEM. Statistical analyses were performed using GraphPad Prism 8.0.2 (GraphPad Software, San Diego, CA, United States). All assays were performed with at least three independent biological replicates. Differences between two groups were analyzed using unpaired two-tailed Student’s t-test. Differences among multiple groups were analyzed using one-way ANOVA followed by Tukey’s multiple comparisons test. SBP measured over time was analyzed using two-way repeated-measures ANOVA followed by Sidak’s multiple comparisons test. P < 0.05 was considered statistically significant.

## 3 Results

### 3.1 PE placentas exhibit enhanced senescence-associated features

**FIGURE 1:**
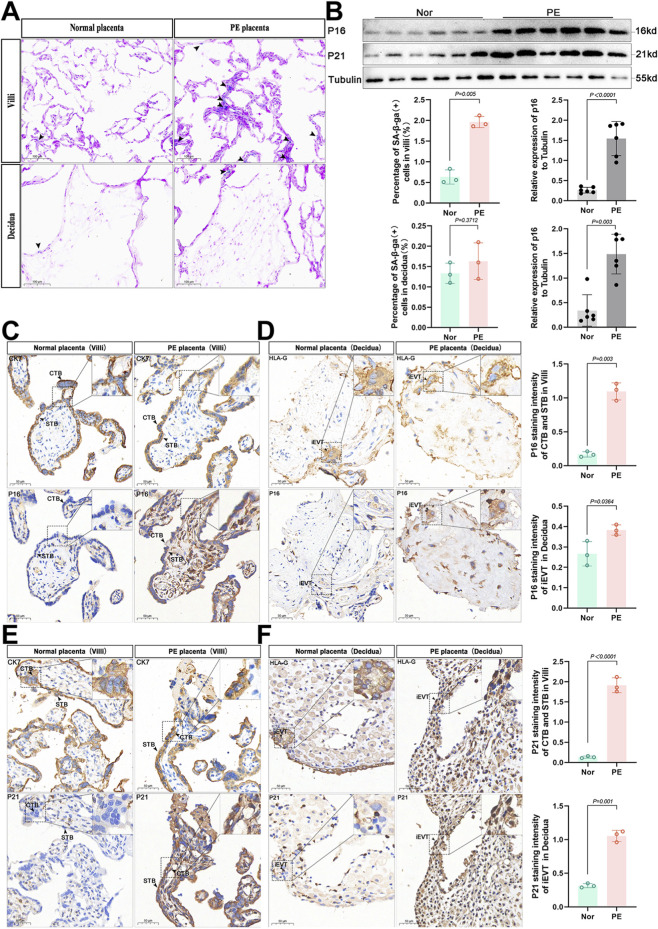
The Placenta of preeclampsia pregnancy is associated with Premature Senescence **(A)** SA-β-gal staining in human PE and normal placentas. Quantification of staining intensity per patient; Scale bars:100 μm; n = 3. **(B)** Western blot analysis of protein expression of p16 and p21 in placenta tissues; n = 6. IHC staining of **(C,D)** p16 and **(E,F)** p21 in human PE and normal placentas. Quantification of staining intensity per patient; Scale bars: 50 μm; n = 3. iEVTs and CTBs were identified by HLA-G and CK7 staining, respectively. STB, syncytiotrophoblasts; iEVT, interstitial extravillous trophoblast; CTBs, cytotrophoblasts; CK7, cytokeratin 7; HLA-G, human leukocyte antigen G. two-tailed t-test. All data are presented as the means ± SEM.

Premature placental senescence has increasingly been implicated in adverse pregnancy outcomes (Scaife et al., 2021). We first investigated whether premature placental senescence occurs in pregnancies complicated by PE. SA-β-gal staining was detectable in both villous and decidual tissues of the placenta, with predominant localization in syncytiotrophoblasts (STBs) and cytotrophoblasts (CTBs) within villous tissues. Compared with normal placentas, PE placentas displayed markedly increased SA-β-gal positivity in villous tissues. In contrast, decidual tissues contained relatively few positive cells, and no obvious difference was observed between the PE and control groups (Figure 1A).

We next further examined the expression of core senescence-associated proteins. Western blot showed that p16 and p21 were both significantly upregulated in PE placentas (Figure 1B). Immunohistochemical staining further demonstrated p16 and p21 expressed in STBs, CTBs, and interstitial extravillous trophoblasts (iEVTs), with substantially stronger staining in PE placentas than in control placentas (Figures 1C–F). These findings indicate that PE placentas, particularly the villous trophoblast region, exhibit enhanced senescence-associated phenotype.

### 3.2 SIRT1 deficiency may contribute to premature placental senescence

Xiong et al. have shown that SIRT1 deficiency promotes premature cellular senescence (Xiong et al., 2021). To further assess the role of SIRT1 in placental senescence, we first examined its expression in placental tissues. Western blot analysis showed that SIRT1 protein levels were markedly reduced in PE placentas compared with normal placentas (Supplementary Figure S1A). Immunohistochemical staining further indicated that SIRT1 was expressed in multiple trophoblast populations, including STBs, CTBs, and iEVTs, whereas its staining intensity was clearly diminished in PE placentas, consistent with the Western blot results (Supplementary Figures S1B–S1C).

**FIGURE 2:**
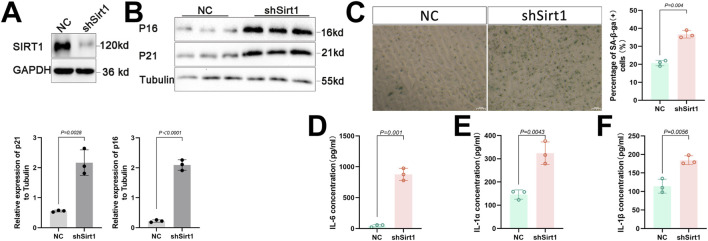
SIRT1 deficiency promotes senescence in trophoblast Cells **(A)** Western blot validates shSIRT1 interference efficiency in HTR8/SVneo cells **(B)** Western blot detection of the protein levels of p16 and p21; n = 3. **(C)** SA-β-gal staining of HTR8/SVneo cells; Scale bars: 200 μm; n = 3. **(D–F)** Levels of **(D)** IL-6, **(E)** IL-1αand **(F)** IL-1β concentration in the cells were measured using the corresponding detection kits; n = 3 independent experiments. Two-tailed t-test. All data are presented as the means ± SEM.

On this basis, we further explored the relationship between SIRT1 loss and premature trophoblast senescence at the cellular level. HTR-8/SVneo cells were subjected to SIRT1 knockdown, and transfection efficiency was confirmed by Western blotting (Figure 2A). Compared with the control group, SIRT1 knockdown significantly increased p16 and p21 protein levels (Figure 2B). The proportion of SA-β-gal-positive cells also increased significantly (Figure 2C), indicating the development of a senescence-like phenotype.

Senescent cells promote disease progression through the secretion of bioactive factors, collectively referred to as the senescence-associated secretory phenotype (SASP) (Wang et al., 2024). Given that senescent cells are often accompanied by enhanced SASP, we next measured related secreted factors. The levels of SASP-associated cytokines, including IL-6, IL-1α, and IL-1β, were significantly elevated in shSIRT1 cells (Figures 2D–F). These findings suggest that SIRT1 is downregulated in PE placentas, and that deficiency of SIRT1 in trophoblasts promotes a senescence-associated phenotype and enhances SASP secretion. Whether this process further contributes to PE progression remains to be determined.

### 3.3 SIRT1 attenuates PE-like phenotypes and ameliorates placental senescence *in vivo*

**FIGURE 3:**
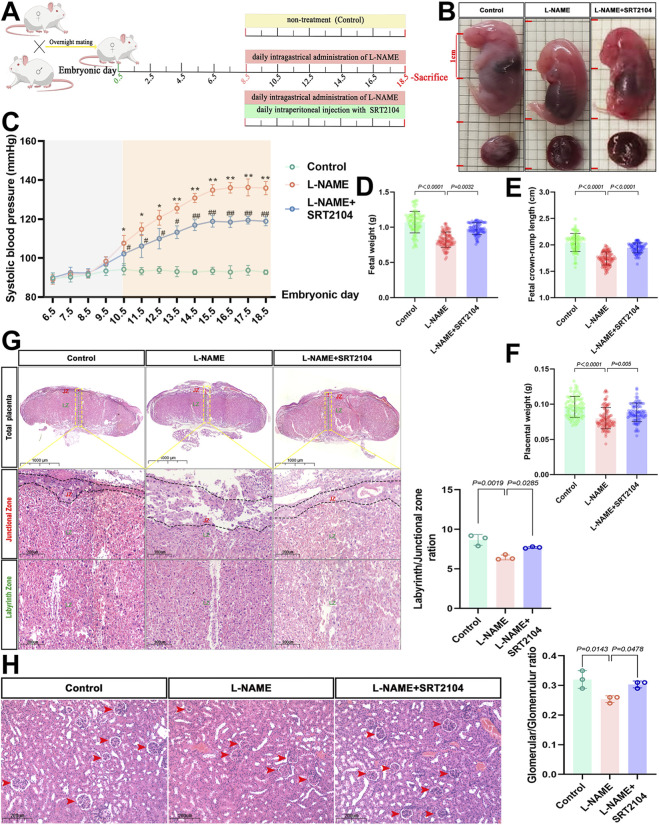
SIRT1 alleviates PE-like symptoms in the mouse model **(A)** Schematic illustration of the experimental design. **(B)** Representative images of the fetuses of the Control, L-NAME, and L-NAME + SRT2104 groups; Scale bars:1 cm. **(C)** Systolic blood pressure of pregnant mice in control (n = 6 dam), L-NAME (n = 7 dams), and L-NAME + SRT2104 group (n = 7 dams). **(D–F)** Fetal weight, **(E)** crown-rump length,and **(F)** placental weight at E18.5 in different groups; n = 85 fetuses from 6 dams in the control group, n = 98 fetuses from 7 dams in the L-NAME group, and n = 81 fetuses from 7 dams in the L-NAME + SRT2104 group. **(G)** H&E staining of placental sections at E18.5. The LZ and JZ areas and the Lz/Jz ratio were quantified; Scale bars: 1000 μm (upper panel), 200um (lower panel); n = 3. **(H)** H&E staining of maternal kidney sections at E18.5, and measurement of Bowman space; Scale bars: 200 μm; n = 3. LZ, Labyrinth zone; JZ, Junction zone. One-way ANOVA and Tukey’s multiple comparison test. All data are presented as the means ± SEM.

To further evaluate the role of SIRT1 in an *in vivo*, we first established a PE-like mouse model by L-NAME administration, as reported previously (de Alwis et al., 2022), and then treated a subset of animals with the SIRT1 agonist SRT2104 (Figure 3A). Compared with the control group, mice treated with L-NAME exhibited a marked increase in mean SBP from E10.5 onward, whereas SRT2104 significantly attenuated this increase (Figure 3C). In terms of pregnancy outcomes, at E18.5, the L-NAME-treated group exhibited significantly reduced fetal weight, placental weight, and crown-rump length compared with controls, and these abnormalities were partially improved by SRT2104 treatment (Figures 3B,D–F). Histological analysis further showed that L-NAME induced structural abnormalities in the placental labyrinth zone and reduced the labyrinth-to-junctional zone ratio, whereas SRT2104 partially restored these morphological changes (Figure 3G). In addition, SRT2104 alleviated L-NAME-induced glomerular constriction in maternal kidneys (Figure 3H).

**FIGURE 4:**
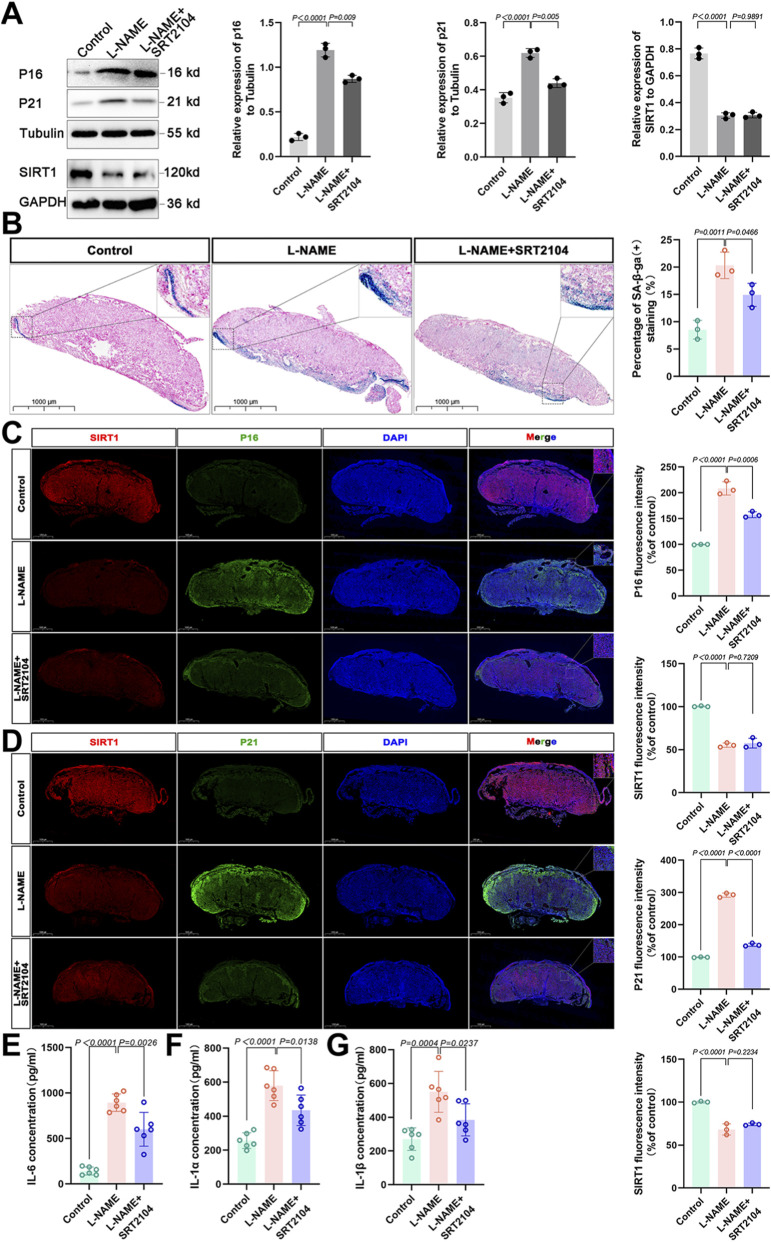
SIRT1 rescues placental senescence in the PE mouse model **(A)** Western blot detection of the protein levels of p16, p21 and SIRT1 in E18.5 mouse placentas from different groups; n = 3. **(B)** SA-β-gal staining in E18.5 mouse placentas from different groups. Scale bars: 1,000 μm, n = 3 **(C,D)** Representative immunofluorescence staining of **(C)** SIRT1(red) and p16 (green) or **(D)** SIRT1 (red) and p21 (green), in E18.5 placentas from different groups; Nuclei were counterstained with DAPI (blue). Scale bars: 1,000 μm; n = 3. **(E–G)** Levels of **(E)**IL-6, **(F)** IL-1α and **(G)** IL-1β concentration in the E18.5 mouse placentas from different groups were measured using the corresponding detection kits; n = 3, one-way ANOVA and Tukey’s multiple comparison test. All data are presented as the means ± SEM.

We next assessed senescence-associated changes in mouse placentas. Western blot analysis showed that L-NAME treatment increased placental p16 and p21 expression, whereas these changes were clearly attenuated by SRT2104 (Figure 4A). SA-β-gal staining yielded similar results, with a clear increase in positive staining in the L-NAME group and a reduction after SRT2104 treatment (Figure 4B). Immunofluorescence co-localization analysis showed that L-NAME reduced SIRT1 expression and increased p16 and p21 expression, whereas SIRT1 activation effectively reversed these changes (Figures 4C,D). In addition, placental levels of SASP-related factors were elevated after L-NAME treatment and decreased following SRT2104 intervention (Figures 4E–G). Taken together, these data indicate that placental senescence is present in the L-NAME-induced PE-like model. Activation of SIRT1 alleviates PE-like manifestations and attenuates placental senescence-associated changes *in vivo*.

### 3.4 SIRT1 loss-associated senescence contributes to trophoblast dysfunction

**FIGURE 5:**
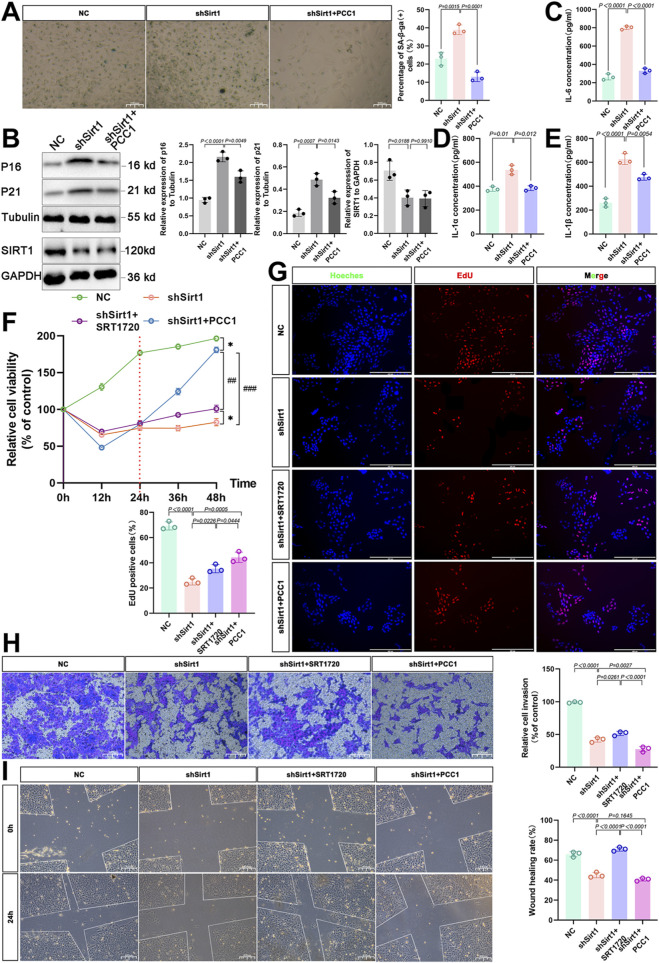
Elimination of senescent cells alleviates SIRT1 deficiency–induced dysfunction and SASP **(A–E)** HTR-8/SVneo cells were transfected with shSIRT1 and subsequently treated with PCC1 (5 μM) for 24 h **(A)** SA-β-gal staining cells; Scale bars: 200 μm; n = 3. **(B)** Western blot analysis of the protein levels of p16, p21 and SIRT1 in cells; n = 3. **(C–E)** Levels of **(C)** IL-6 **(D)** IL-1α and **(E)** IL-1β concentration in the cells were measured using corresponding detection kits; n = 3. **(F)** HTR-8/SVneo cells were transfected with shSIRT1 and then treated with PCC1 (5 μM) or SRT1720 (0.4 uM) for various time periods. Cell viability was measured by CCK8; n = 6. **(G–I)** HTR-8/SVneo cells were transfected with shSIRT1 and subsequently treated with PCC1 (5 μM) or SRT1720 (0.4 uM) for 24 h. **(G)** EdU staining; Scale bars: 400 μm; n = 3 **(H)** Matrigel Transwell assay; Scale bars: 200 μm; n = 3. **(I)** Wound-healing assay; Scale bars: 400 μm; n = 3. One-way ANOVA and Tukey’s multiple comparison test. All data are presented as the means ± SEM.

Given that SIRT1 deficiency induced a senescence-like phenotype in trophoblasts, we next examined the relationship between senescence and trophoblast function. Procyanidin C1 (PCC1), a senescence-targeting compound, was added to shSIRT1-treated HTR-8/SVneo cells (Xu et al., 2021). SA-β-gal staining showed that PCC1 reduced the proportion of SA-β-gal-positive cells (Figure 5A). Western blotting further showed that PCC1 attenuated the shSIRT1-induced increase in p16 and p21 without affecting SIRT1 expression itself (Figure 5B). In parallel, PCC1 also reduced the extracellular levels of SASP-associated factors, including IL-6, IL-1α, and IL-1β (Figures 5C–E).

We then assessed changes in trophoblast function. CCK-8 assays showed that both the SIRT1 agonist SRT1720 and PCC1 partially rescued the decrease in cell viability caused by SIRT1 knockdown. Notably, PCC1 further reduced cell viability during the first 24 h of treatment, whereas this effect was markedly attenuated thereafter (Figure 5F). Functional assays showed that shSIRT1 significantly suppressed HTR-8/SVneo cell proliferation (Figure 5G), invasion (Figure 5H), and migration (Figure 5I). As expected, SIRT1 activation substantially improved all three phenotypes. By contrast, PCC1 improved cell proliferation but did not consistently rescue invasion or migration, and instead exerted a modest inhibitory effect on both processes (Figures 5G–I). Together, these findings indicate that SIRT1 deficiency-associated senescence contributes to trophoblast dysfunction and enhanced SASP, while changes in invasion and migration may also involve mechanisms beyond senescence alone.

### 3.5 Iron dyshomeostasis correlates with SIRT1 deficiency

**FIGURE 6:**
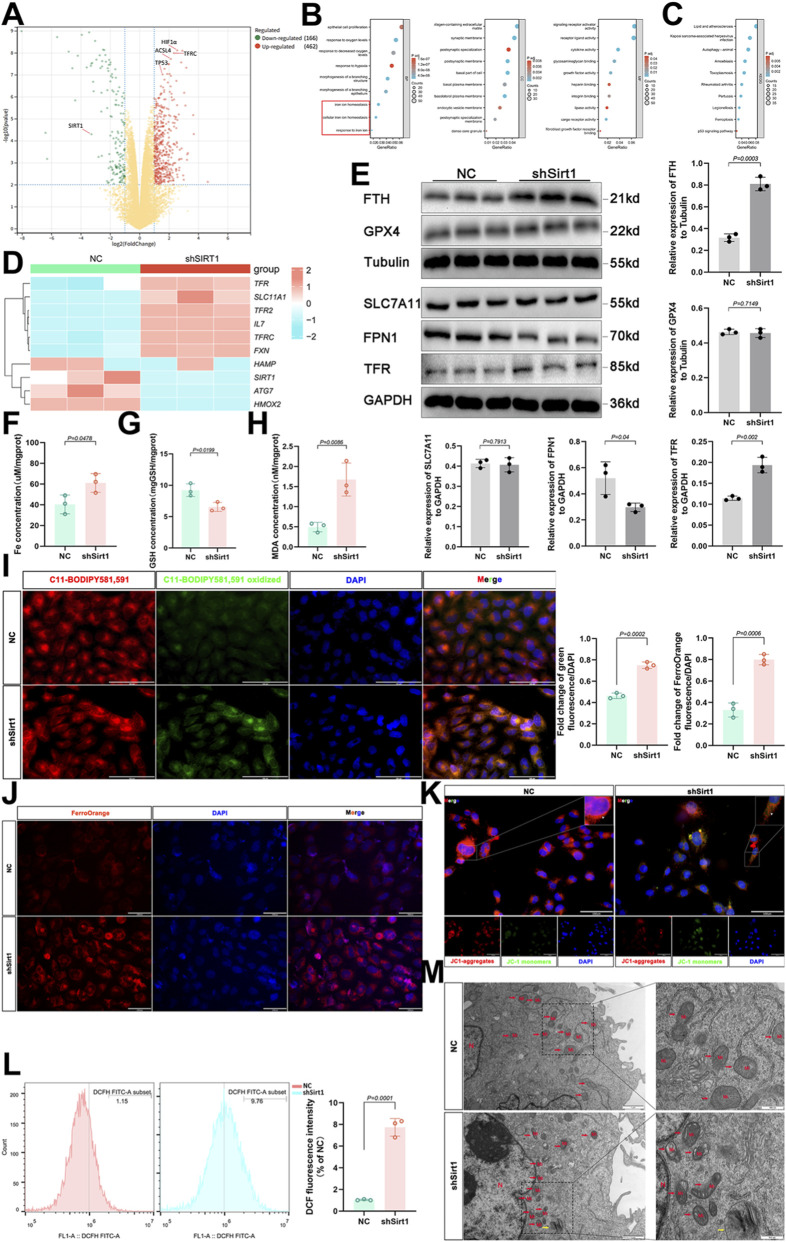
Iron metabolism dysregulation correlates with SIRT1 deficiency **(A)** Volcano plot of the significant differences in gene expression levels between groups. Genes showing the greatest differences were analyzed by **(B)** GO and **(C)** KEGG analysis. **(D)** Heatmap depicting the expression levels of representative genes associated with “intracellular iron ion homeostasis”. **(E)** Western blot detection of the protein levels of FTH, GPX4, SLC7A11, FPN1 and TFR; n = =3. **(F–H)** Measurement of intracellular **(F)** iron concentration, **(G)** GSH concentration, and **(H)** MDA concentration using corresponding detection kits; n = 3. **(I)** Measuring cellular lipid peroxidation by fluorescence microscopy using the C11 BODIPY 581/591 fluorescent probe. Total C11 BODIPY 581/591 (red), oxidized C11 BODIPY 581/591 (green), DAPI (blue) stained nucleus; Scale bar: 100 μm; n = 3. **(J)** Intracellular Fe^2+^ was measured by fluorescence microscopy using the FerroOrange fluorescent probe. FerroOrange (red) stains Fe^2+^ and DAPI (blue) stains the nucleus; Scale bar: 200 μm; n = 3. **(K)** Measuring mitochondrial membrane potential using the JC-1 fluorescent probe. JC-1 aggregates (red), JC-1 monomers (green) and DAPI (blue) stained nucleus; Scale bar: 100 μm; n = 3. **(L)** Flow cytometry assay for measuring ROS by staining with DCFH-DA, normalized by the number of cells uploaded; n = 3 **(M)** Transmission electron microscopy images of organelles. Scale bars: 1 µm (left panel) and 500 nm (right panel); N, nucleus; Mi, mitochondria. N = 3, two-tailed t-test. All data are presented as the means ± SEM.

To further investigate the mechanisms by which SIRT1 deficiency impairs trophoblast function, we performed transcriptomic sequencing in shSIRT1-treated HTR-8/SVneo cells. Compared with the controls, 628 differentially expressed mRNAs were identified, including 462 upregulated and 166 downregulated transcripts (P < 0.01, FC > 2) (Figure 6A). GO analysis showed enrichment in biological processes related to iron metabolism, oxidative stress, and cell growth (Figure 6B; Supplementary Table S4). KEGG analysis further revealed enrichment in pathways including p53 signaling, autophagy, and lipid and atherosclerosis (Figure 6C; Supplementary Table S5).

We next focused on the relationship between SIRT1 deficiency and iron dyshomeostasis. By integrating the FerrDb and GO databases and using “intracellular iron ion homeostasis” as the search term, we identified 84 candidate genes (Supplementary Table S6). Among these, TFR, SLC11A1, and TFR2 were markedly downregulated after SIRT1 deficiency, whereas HAMP, ATP7, and HMOX2 were upregulated (Figure 6D). We then examined proteins involved in iron metabolism and antioxidant defense. SIRT1 knockdown caused clear alterations in iron storage- and transport-related proteins, including FTH, TFR, and FPN1, whereas no obvious changes were detected in GPX4 or SLC7A11, two core components of the antioxidant defense axis (Figure 6E).

We next validated intracellular iron load and oxidative stress. SIRT1 deficiency increased Fe^2+^ levels in trophoblasts (Figures 6F,J), elevated MDA (Figure 6H) and ROS levels (Figure 6L), reduced GSH levels (Figure 6G), and enhanced lipid peroxidation (Figure 6I). Mitochondrial dysfunction is a typical consequence of iron dyshomeostasis. Consistently, mitochondrial membrane potential was reduced (Figure 6K), and transmission electron microscopy (TEM) revealed shrunken mitochondria with reduced or lost cristae (Figure 6M), indicating mitochondrial injury.

To assess whether this iron-related phenotype was also present *in vivo*, we examined placentas from the L-NAME-induced PE-like mouse model. Perls’ blue staining showed a significant increase in iron-positive cells in placentas from the L-NAME group (Supplementary Figure S3A). Consistently, SRT2104 treatment reduced placental iron and MDA levels while increasing GSH levels (Supplementary Figures S3C–S3E). Immunohistochemistry further showed that SRT2104 partially restored the abnormal expression of FTH, TFR, and FPN1 induced by L-NAME (Supplementary Figure S3B).

In addition, we analyzed an external dataset (GSE100279) to further support the pathway alterations associated with SIRT1 dysregulation (Arul Nambi Rajan et al., 2018). GO and KEGG analyses again showed enrichment of genes associated with iron uptake and related processes (Supplementary Figures S2A–S2B; Supplementary Table S7). Taken together, these findings indicate that SIRT1 deficiency is accompanied by iron dyshomeostasis, oxidative injury, and lipid peroxidation in trophoblasts and PE placentas.

### 3.6 Iron dyshomeostasis drives a senescence-associated feedback loop

**FIGURE 7:**
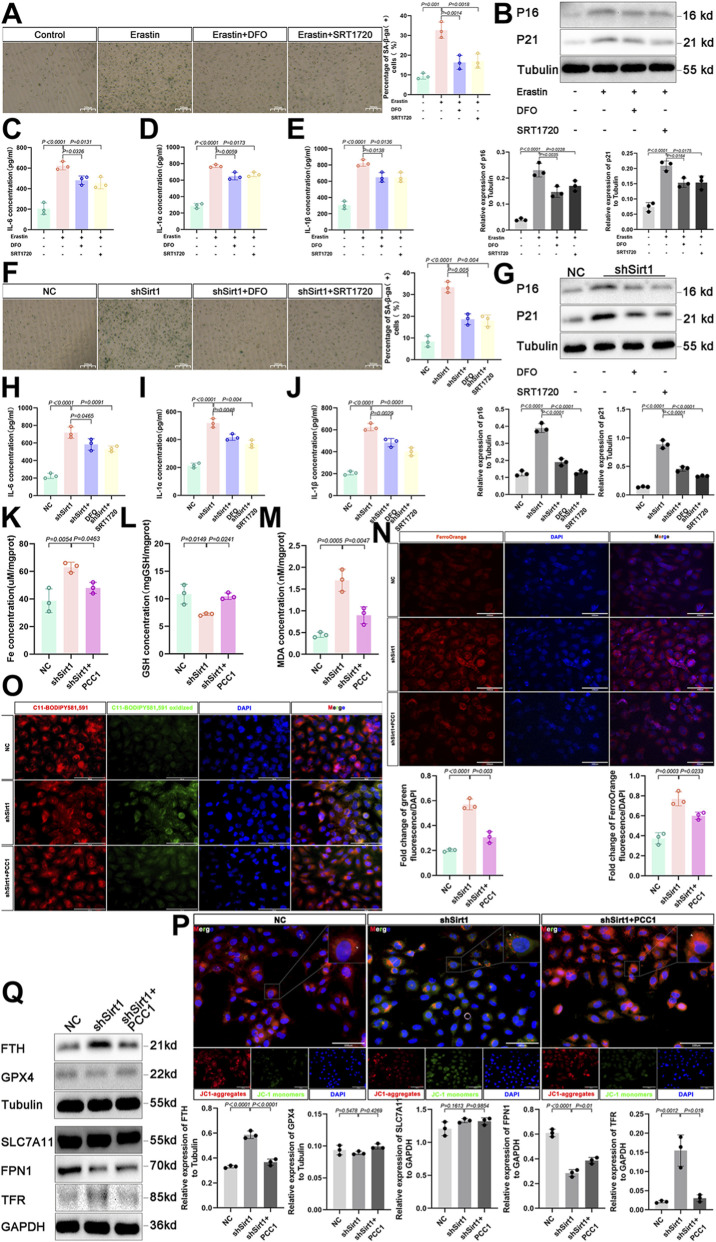
Iron metabolism dysregulation drives senescence feedback loop **(A–E)** Cells were pretreated with the iron chelator deferoxamine (DFO, 100 µM) or SRT1720 (0.4 µM) for 1 h, followed by 24-h treatment with Erastin (5 µM). **(A)** SA-β-gal staining cells; Scale bars: 200 μm; n = 3. **(B)** Western blot analysis of the protein levels of p16 and p21 in cells; n = 3. **(C–E)** Levels of **(C)** IL-6, **(D)** IL-1α and **(E)** IL-1β concentration in the cells were measured using corresponding detection kits; n = 3. **(F–J)** HTR-8/SVneo cells were transfected with shSIRT1 and then treated with DFO (100 μM) or SRT1720 (0.4 uM) for 24 h. **(F)** SA-β-gal staining cells; Scale bars: 200 μm; n = 3. **(G)** Western blot analysis of the protein levels of p16 and p21 in cells; n = 3. **(H–J)** Levels of **(H)** IL-6, **(I)** IL-1α and **(J)** IL-1β concentration in cells were measured using corresponding detection kits; n = 3. **(K–Q)** HTR-8/SVneo cells were transfected with shSIRT1 and then treated with PCC1 (5 μM) for 24 h. **(K–M)** Measurement of **(K)** intracellular iron concentration, **(I)** GSH concentration, and **(M)** MDA concentration using corresponding detection kits; n = 3. **(N)** Measuring intracellular Fe^2+^. FerroOrange (red) stains Fe^2+^ and DAPI (blue) stains the nucleus; Scale bar: 200 μm; n = 3. **(O)** Measuring cellular lipid peroxidation. Total C11 BODIPY 581/591 (red), oxidized C11 BODIPY 581/591 (green), DAPI (blue) stained nucleus; Scale bar: 100 μm; n = 3. **(P)** Measuring mitochondrial membrane potential. JC-1 aggregates (red), JC-1 monomers (green) and DAPI (blue) stained nucleus; Scale bar: 100 μm; n = 3. **(Q)** Western blot detection of the protein levels of FTH, GPX4, SLC7A11, FPN1 and TFR; n = 3. One-way ANOVA and Tukey’s multiple comparison test. All data are presented as the means ± SEM.

Maus et al. reported a close association between dysregulated iron metabolism and senescence-related phenotypes (Maus et al., 2023). We next asked whether iron dyshomeostasis and senescence are functionally linked in trophoblasts. HTR-8/SVneo cells were treated with Erastin to induce an iron-overloaded intracellular environment (Yang et al., 2022). Erastin markedly increased the proportion of senescent trophoblasts, and this effect was effectively reversed by either the iron chelator deferoxamine (DFO) or the SIRT1 agonist SRT1720 (Figure 7A). Western blotting showed that Erastin increased p16 and p21 expression, whereas both iron chelation and SIRT1 activation partially restored these changes (Figure 7B). In addition, Erastin-induced SASP secretion was attenuated by DFO or SRT1720 (Figures 7C–E). These findings indicate that iron dyshomeostasis is sufficient to promote a senescence-associated phenotype in trophoblasts.

We then examined the reverse relationship. In the shSIRT1-induced senescent HTR-8/SVneo model, treatment with DFO or SRT1720 reduced cellular senescence (Figures 7F,G) and partly suppressed SASP secretion (Figures 7H–J). These results suggest that iron dyshomeostasis contributes to the senescence process induced by SIRT1 deficiency.

To further test whether senescence itself influences iron homeostasis, we treated senescent shSIRT1 cells with PCC1 and evaluated iron-related changes. Inhibition of senescence reduced intracellular iron levels (Figures 7K,N), lowered MDA levels (Figure 7M), increased GSH levels (Figure 7L), and attenuated lipid peroxidation (Figure 7O). PCC1 also partially restored mitochondrial membrane potential (Figure 7P). Western blotting analysis showed that inhibition of senescence markedly altered the expression of FTH, FPN1, and TFR, whereas GPX4 and SLC7A11 remained largely unchanged (Figure 7Q). Collectively, these findings further support a pathogenic role for SIRT1 dysregulation in trophoblasts by driving a positive feedback loop between senescence and iron dyshomeostasis, consistent with a ferro-aging-like state.

### 3.7 The SIRT1–p53 axis mediates iron homeostasis and senescence in trophoblasts

**FIGURE 8:**
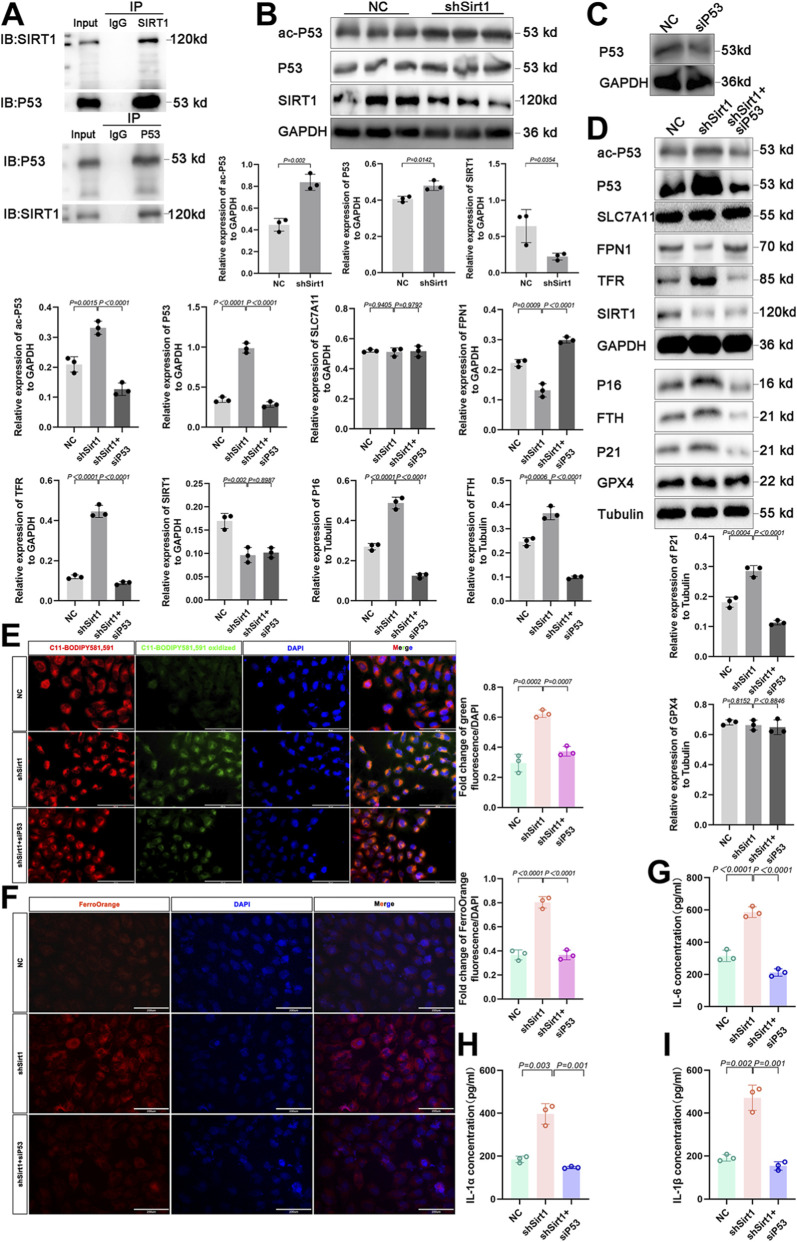
SIRT1 regulates iron metabolism and senescence in trophoblast cells via p53 deacetylation **(A)** Reciprocal Co-IP of SIRT1 and p53 in HTR8/SVneo cells. **(B)** Western blot analysis of ac-p53, p53, and SIRT1; n = 3. **(C)** Western blot validates si-p53 interference efficiency. **(D)** Western blot detection of the protein levels of ac-p53, p53, SLC7A11, FPN1, TFR, SIRT1, p16, FTH, p21 and GPX4; n = 3. **(E)** After treatment, fluorescence images of total C11 BODIPY 581/591 (red) and oxidized C11 BODIPY 581/591 (green) in HTR-8/SVneo cells as indicated; Scale bar: 100 μm; n = 3. **(F)** After treatment, fluorescence images of Fe^2+^(red) in HTR-8/SVneo cells as indicated; Scale bar: 200 μm; n = 3. **(G–I)** Levels of **(G)** IL-6, **(H)** IL-1α and **(I)** IL-1β concentration in cells were measured using corresponding detection kits; n = 3. One-way ANOVA and Tukey’s multiple comparison test. All data are presented as the means ± SEM.

SIRT1 is a well-established deacetylase of p53, and SIRT1 deficiency has been associated with p53 hyperacetylation and enhanced senescence-related signaling (Cheng et al., 2003; Ong and Ramasamy, 2018). To explore the downstream mechanism of SIRT1, we first performed reciprocal co-immunoprecipitation in HTR-8/SVneo cells and confirmed an interaction between SIRT1 and p53 (Figure 8A). Further analysis showed that SIRT1 knockdown markedly increased acetylated p53 levels, whereas total p53 protein remained unchanged (Figure 8B), indicating that SIRT1 downregulation is associated with enhanced p53 acetylation.

We next asked whether p53 mediates the effects of SIRT1 deficiency on iron homeostasis and senescence. p53 was silenced in SIRT1-deficient HTR-8/SVneo cells, and knockdown efficiency was confirmed by Western blotting (Figure 8C). Compared with the control group, p53 knockdown reduced p53 acetylation to some extent and reversed the SIRT1 deficiency-induced changes in the iron homeostasis-related proteins FPN1, TFR, and FTH. At the same time, the levels of the senescence markers p16 and p21 were also decreased (Figure 8D).

We then evaluated the effects of p53 knockdown on iron metabolism and senescence-associated phenotypes. p53 silencing reduced the increase in lipid peroxidation induced by SIRT1 deficiency (Figure 8E), decreased intracellular iron accumulation (Figure 8F) and partially lowered the secretion of SASP-associated factors, including IL-6, IL-1α, and IL-1β (Figures 8G–I). Together, these findings indicate that SIRT1 downregulation is accompanied by enhanced p53 acetylation, and that p53 silencing partially reverses the iron homeostasis abnormalities and senescence-associated changes induced by SIRT1 deficiency. These data place the SIRT1–p53 axis upstream of the coupled iron dyshomeostasis–senescence program in trophoblasts.

## 4 Discussion

PE remains a major cause of adverse maternal and perinatal outcomes, with placental dysfunction at being central to its pathogenesis and the ensuing maternal systemic response (Dimitriadis et al., 2023). Recent studies have integratedplacental senescence into the “two-stage model” of PE, highlighting how ischemia-hypoxia, oxidative stress, mitochondrial injury, and inflammatory stimuli may accelerate premature placental aging, amplify placenta-derived pathogenic signals, and ultimately contributing to maternal hypertension and endothelial injury (Staff, 2019; Sugulle et al., 2024). Within this framework, our study supportsthe concept placental senescence is not a mere secondary epiphenomenon, but rather a biologically meaningful component of PE pathology. By integrating clinical placental samples, trophoblast-based experiments, and an L-NAME-inducedPE-like mouse model, we identified a coherent pathogenic axis characterized by reduced SIRT1 expression, enhanced senescence-associated changes, iron dyshomeostasis, lipid peroxidation, and increased p53 acetylation. These findings place the SIRT1–p53 axis upstream of a coupled iron dyshomeostasis–senescence program in PE placentas.

Placental senescence itself is not inherently pathological. During normal gestation, the placenta undergoes a degree of physiological senescence-like change, associated with trophoblast fusion, tissue maturation, and sterile inflammatory activation near term (Cox and Redman, 2017). The problem arises when this process occurs prematurely or becomes excessively amplified. Under such conditions, senescence may shift from adaptive remodelling to pathological injury and contribute to placental insufficiency, fetal growth restriction, and PE (Roh et al., 2024). In the present study, we observed that SA-β-gal, p16, and p21 were markedly increased in PE placentas and were predominantly localized to the villous trophoblast compartment. This distribution pattern is more consistent with pathological premature senescence, rather senescence associated with normal gestational progression. Notably, although the PE group delivered at an earlier gestational age than the control group and would therefore be expected to exhibit fewer physiological senescence features, senescence markers were more pronounced in PE placentas. This finding supports the possibility that PE placentas enter a senescent state prematurely. We speculate that chronic placental stress, including hypoxia-reoxygenation and reperfusion injury, may drive early senescence in PE (Negre-Salvayre et al., 2022; Redman et al., 2022). This interpretation still requires validation in an independent gestational age-matched cohort. The increase in placental SASP factors in the PE mouse model further suggests that placental senescence is not merely a consequence of gestational progression but may be intensified under pathological stress and actively contribute to PE pathophysiology.

Our data also reveal a clear spatial bias in this process. Senescence-associated changes were primarily observedin villous trophoblasts rather than in the decidua. This finding is biologically plausible, as villous trophoblasts, especially STBs and CTBs, directly mediate maternal-fetal exchange, maintain redox balance, and contribute to the secretion of placenta-derived factors. These cells are among the first placental compartments exposed to abnormal perfusion, hypoxia-reoxygenation, and lipid peroxidation in PE (Tasta et al., 2021; Redman et al., 2022). By contrast, decidual senescence may play distinct roles across pregnancy disorders. Previous work has suggested that premature senescence in the decidua is more closely linked to early pregnancy loss than to PE as a dominant histopathological feature (de la Torre et al., 2021). Our observations are in line with this interpretation and suggest that senescence in PE is not uniformly distributed, but instead shows marked tissue specificity.

Within this context, SIRT1 emerges as a plausible upstream regulator. SIRT1 is an NAD + -dependent deacetylase with established roles in metabolic homeostasis, oxidative stress responses, mitochondrial function, and cellular senescence (Liu et al., 2022). In the placenta, SIRT1 is considered a key regulator of trophoblast development and function. Previous work has shown that reduced SIRT1 expression is associated not only with abnormal placental development but also with premature placental functional decline (Xiong et al., 2021; Wang et al., 2022). In our study, we found that SIRT1 was abundantly expressed in STBs, CTBs, and iEVTs, but was markedly reduced in PE placentas. In HTR-8/SVneo cells, SIRT1 knockdown increased p16 and p21 expression, enhanced SA-β-gal positivity, and promoted SASP secretion. These findings are consistent with the idea that reduced SIRT1 is not simply a marker of placental stress, but may participate directly in the establishment of a senescence-prone trophoblast state.

The *in vivo* data strengthened this interpretation. We established a PE-like mouse model using L-NAME (McCarthy et al., 2011; de Alwis et al., 2022), a nitric oxide synthase inhibitor and induces PE-like features in pregnant mice during mid-to-late gestation, including hypertension, proteinuria, renal injury, and fetal growth restriction (de Alwis et al., 2022). In this model, we observed both placental senescence and placental structural abnormalities. Przybyl et al. proposed that the placental labyrinth is the principal site of fetal nutrient exchange (Przybyl et al., 2016). In our study, the labyrinth-to-junctional zone ratio was reduced in L-NAME-treated mice, whereas SRT2104 partially restored this ratio and alleviated senescence-associated changes. These findings indicate that pharmacological activation of SIRT1 improves both maternal and placental phenotypes, supporting the view that SIRT1 functions as an upstream regulator rather than a passive marker. Therefore, our data support a close link between SIRT1 deficiency and trophoblast senescence, and suggest that SIRT1 may alleviate PE by attenuating premature placental senescence and improving placental function.

The relationship between low SIRT1 expression and impaired trophoblast behaviour has been explored in previous studies on placental and pregnancy-related disorders (Xiong et al., 2021). Our results show that SIRT1 insufficiency not only induces trophoblast senescence and SASP activation but also impairs proliferation, invasion, and migration. SIRT1 activation effectively reverses both the senescence-associated phenotype and trophoblast dysfunction. We also observed an interesting effect of PCC1. PCC1 reduced SA-β-gal positivity, lowered p16/p21 expression, and suppressed SASP, but did not consistently restore invasion or migration, and transiently reduced cell viability early after treatment. PCC1 is a plant-derived anti-senescence compound that reduces SA-β-gal and p53-related senescence features *in vivo*, while also reshaping tissue and cellular states (Xu et al., 2021). Xu et al. further showed that PCC1 is a time- and dose-dependent apoptosis inducer and senolytic agent. At lower doses, it acts primarily as a senomorphic compound by suppressing SASP, whereas at higher doses it selectively eliminates senescent cells and can induce their death through mitochondrial ROS and membrane potential changes (Xu et al., 2021). Our findings are consistent with this model and suggest that PCC1 reduces the overall senescence burden mainly by selectively clearing senescent cells, rather than reversing senescence itself. Taken together, these results indicate that SIRT1 deficiency-associated senescence is more directly linked to impaired proliferation and enhanced inflammatory secretion, while changes in invasion and migration may involve additional mechanisms, such as cytoskeletal remodelling, epithelial–mesenchymal transition (EMT)-like programs, or metabolic disturbances.

To further investigate how SIRT1 regulates trophoblast function, we performed transcriptome sequencing and identified a significant link between SIRT1 and iron metabolism. Iron metabolism plays a critical role in various pathological processes (Zhang et al., 2022; Chen et al., 2023), with iron disorders encompassing both iron deficiency and iron overload. Our experiments revealed that SIRT1 knockdown led to an increase in intracellular Fe^2+^, a reduction in GSH, and elevated levels of MDA and ROS, alongside loss of mitochondrial membrane potential and mitochondrial ultrastructural abnormalities. These findings are consistent with the emerging model of a “labile iron accumulation–lipid peroxidation–mitochondrial injury” axis in pregnancy-related diseases (Yang et al., 2022; Zhang et al., 2022). Placental iron homeostasis depends on a tightly regulated network: TFR mediates transferrin-bound iron uptake (Chen Y. et al., 2022), FTH buffers iron storage (Fang et al., 2020), and FPN mediates iron export (Bao et al., 2021). Disruption of any of these processes can expand the labile iron pool and amplify ROS generation through the Fenton reaction (Zhang et al., 2022; Yang et al., 2026).

Importantly, our findings argue against a simple interpretation in terms of classical ferroptosis. Although SIRT1 deficiency increased intracellular iron and lipid peroxidation, and altered the expression of FTH, TFR, and FPN1, but did not significantly change GPX4 or SLC7A11, two canonical components of the antioxidant defense axis. Similar patterns were also observed in the external dataset GSE100279. These observations are consistent with Yang et al. (2026), who showed that iron overload-mediated trophoblast dysfunction is an important mechanism in PE. This view is also in line with recent studies suggesting that iron metabolism abnormalities and ferroptosis intersect in PE, although elevated iron levels and lipid peroxidation do not necessarily signify a complete ferroptotic response (Zhang et al., 2022).

Recent work has proposed ferro-aging as a chronic iron-triggered, lipid peroxidation-dependent senescence program that is mechanistically distinct from acute ferroptosis and centered on ACSL4-driven membrane remodelling (Liu et al., 2026). In this context, our results are conceptually aligned with an iron-dependent senescence-promoting state in trophoblasts. However, because ACSL4 was not directly interrogated in this study, we conservatively interpret our findings as supporting a ferro-aging-like process in PE placentas, rather than claiming definitive ACSL4-dependent ferro-aging.

Accumulating evidence implicates dysregulated iron metabolism–induced iron overload as a key driver of pathological senescence and fibrosis (Maus et al., 2023; Amengual et al., 2024). Our results indicate that iron dyshomeostasis and senescence are not arranged in a linear sequence, but instead reinforce each other. Erastin-induced iron overload was sufficient to increase senescence markers and SASP, whereas iron chelation and SIRT1 activation attenuated these changes. This suggests that iron-dependent oxidative stress is sufficient to intensify the senescence program. Conversely, in the senescence model induced by SIRT1 knockdown, inhibition of senescence reduced intracellular iron, attenuated lipid peroxidation, improved mitochondrial membrane potential, and altered iron-handling proteins. These bidirectional effects support the existence of a self-reinforcing interaction between iron dyshomeostasis and senescence in trophoblasts. Based on current understanding of senescence-associated metabolic reprogramming, we speculate that senescent cells undergo broad shifts in mitochondrial function, redox balance, and secretory activity, thereby altering iron utilization, storage, and export. Increased labile iron then further amplifies ROS and lipid peroxidation, stabilizes cell-cycle arrest, and sustains SASP release. In this sense, SIRT1 insufficiency appears to promote a feed-forward pathogenic process in which disturbed iron homeostasis and senescence progressively amplify one another.

SIRT1 is a canonical deacetylase of p53, and its restraining effect on p53 activity has been widely established in senescence, inflammation, and stress responses (Cheng et al., 2003; Lee and Gu, 2013). Our data show that SIRT1 loss increased p53 acetylation, and that p53 inhibition partially reverses iron-handling proteins, intracellular iron accumulation, lipid peroxidation, and senescence-associated markers induced by SIRT1 deficiency. These findings suggest that p53 is likely an important downstream effector of SIRT1 and participates in the development of iron homeostasis abnormalities and senescence induced by SIRT1 insufficiency. Mechanistically, increased p53 acetylation appears to contribute to the iron dyshomeostasis- and senescence-associated phenotype downstream of SIRT1 deficiency.

While the present study provides several important findings, several issues remain unresolved and warrant further investigation. One major concern is the difference in gestational age between the PE and control groups. Although the stronger senescence phenotype observed in earlier-gestation PE placentas supports the possibility of premature senescence, gestational age itself remains a confounding factor and should be controlled in future validation cohorts, ideally with larger sample sizes. In addition, our mechanistic analyses relied largely on cell-based systems and the L-NAME mouse model. These models are informative, but they cannot fully recapitulate the spatial heterogeneity of the human placenta, nor can they completely mimic the immune milieu or the hemodynamic complexity that are characteristic of PE. Future studies incorporating single-cell transcriptomics, spatial transcriptomics, or laser capture microdissection may provide a more precise map of SIRT1, iron homeostasis markers, and senescence-associated signals across STBs, CTBs, and EVTs. While our data support a ferro-aging-like process, definitive classification of this program will require direct evaluation of ACSL4-centered lipid remodelling and other ferro-aging regulatory nodes (Liu et al., 2026). Finally, although our results support a role for SIRT1–p53 signalling in trophoblast dysfunction, p53 knockdown reduced total p53 abundance as well as acetylation. We therefore cannot yet distinguish the specific contribution of p53 acetylation from that of overall p53 loss. This issue will require additional studies using site-specific mutants or more selective pharmacological approaches.

## 5 Conclusion

**FIGURE 9:**
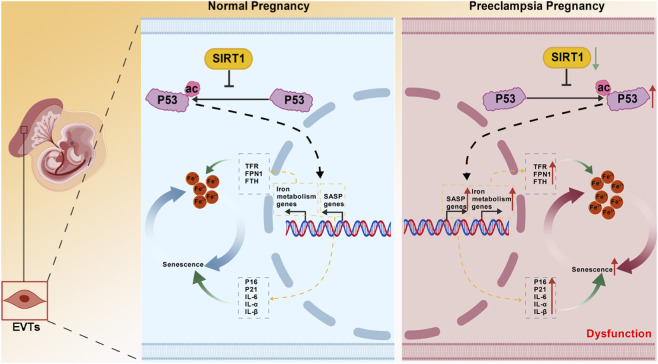
Schematic illustration of the mechanism by which SIRT1 drives a positive feedback loop between iron metabolism and senescence in trophoblasts.

In summary, our study identifies the SIRT1–p53 axis as an upstream regulator linking iron dyshomeostasis to trophoblast senescence in preeclampsia. Reduced SIRT1 expression appears to aggravate placental dysfunction by promoting senescence-associated changes together with iron dyshomeostasis, lipid peroxidation, and oxidative stress (Figure 9). These findings support a ferro-aging-like process in PE placentas and highlight SIRT1-related pathways as potential therapeutic targets. Further spatial omics and single-cell analyses will help clarify this program across trophoblast populations and assess the translational potential of SIRT1-targeted interventions.

## Data Availability

The datasets presented in this study are deposited in the SRA repository. The names of the repository/repositories and accession number(s) can be found below: PRJNA1439633.
