# Supplementary material for: The SIRT1–p53 axis drives a ferro-aging-like program and aggravates trophoblast dysfunction in preeclampsia
Source: Front Aging. 2026 Jun 3;7:1838730. doi: 10.3389/fragi.2026.1838730 (PMC13272158; doi:10.3389/fragi.2026.1838730)
Supplement: Supplementary file 1 [file Table1.docx]

Supplementary Material

**Figure S1. Downregulation of SIRT1 expression in the placenta of PE pregnancies**


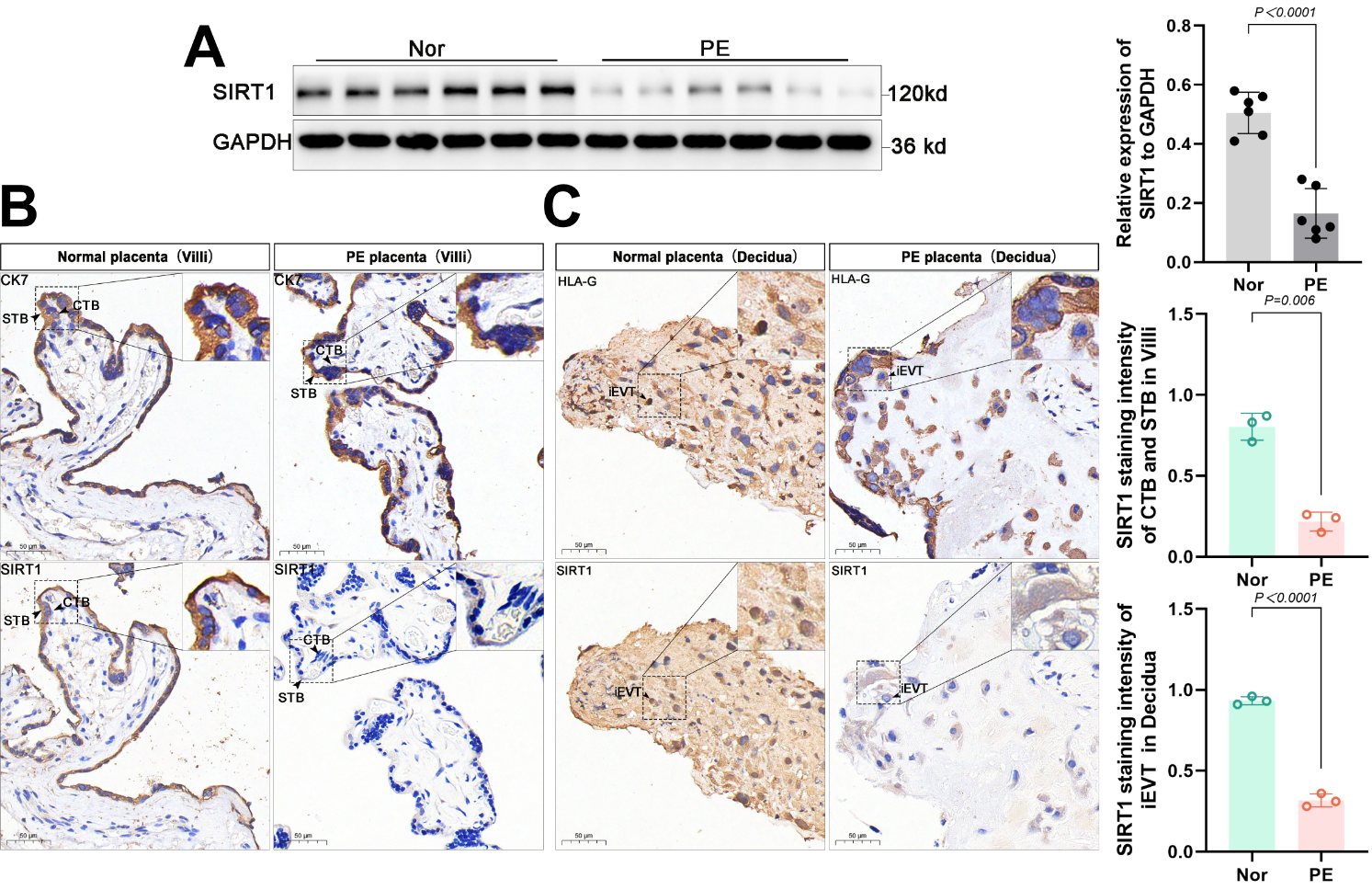


**(a)** Western blot analysis of protein expression of SIRT1 in placenta tissues; n=6. **(b，c)** IHC staining and quantification of SIRT1 expression in villous tissues **(b)** and decidual tissues **(c)** from normal and preeclamptic placentas. Scale bars: 50 μm; n=3, two-tailed t-test. All data are presented as the means ± SEM.

**Figure S2.** **Bioinformatic analysis of GSE100279 dataset**


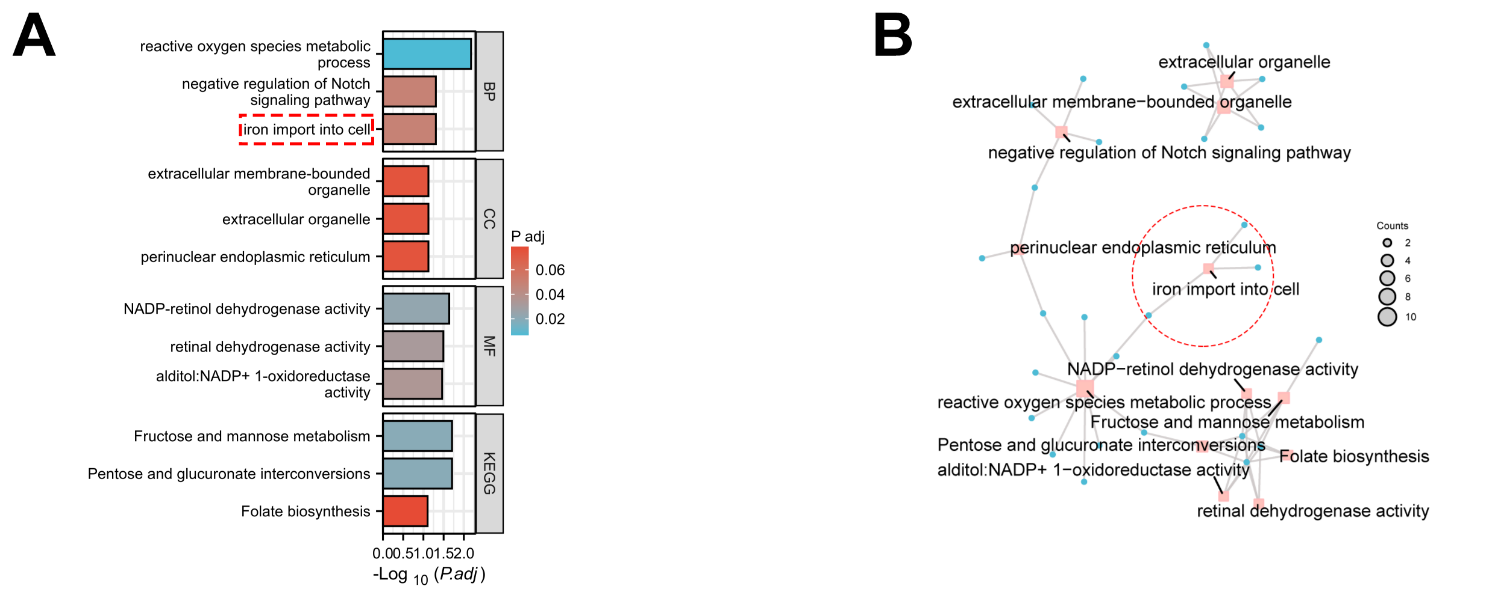


**Figure S2.** **Bioinformatic analysis of GSE100279 dataset**

GO and KEGG pathway enrichment analyses of GSE100279. **(a)** Bar plot illustrating the most significantly enriched GO and KEGG terms，and **(b)**Network visualization of the pathways identified in both GO and KEGG analyses.

**Figure S3.** **SIRT1 rescues iron metabolism dysregulation in the placenta of preeclampsia mouse model**


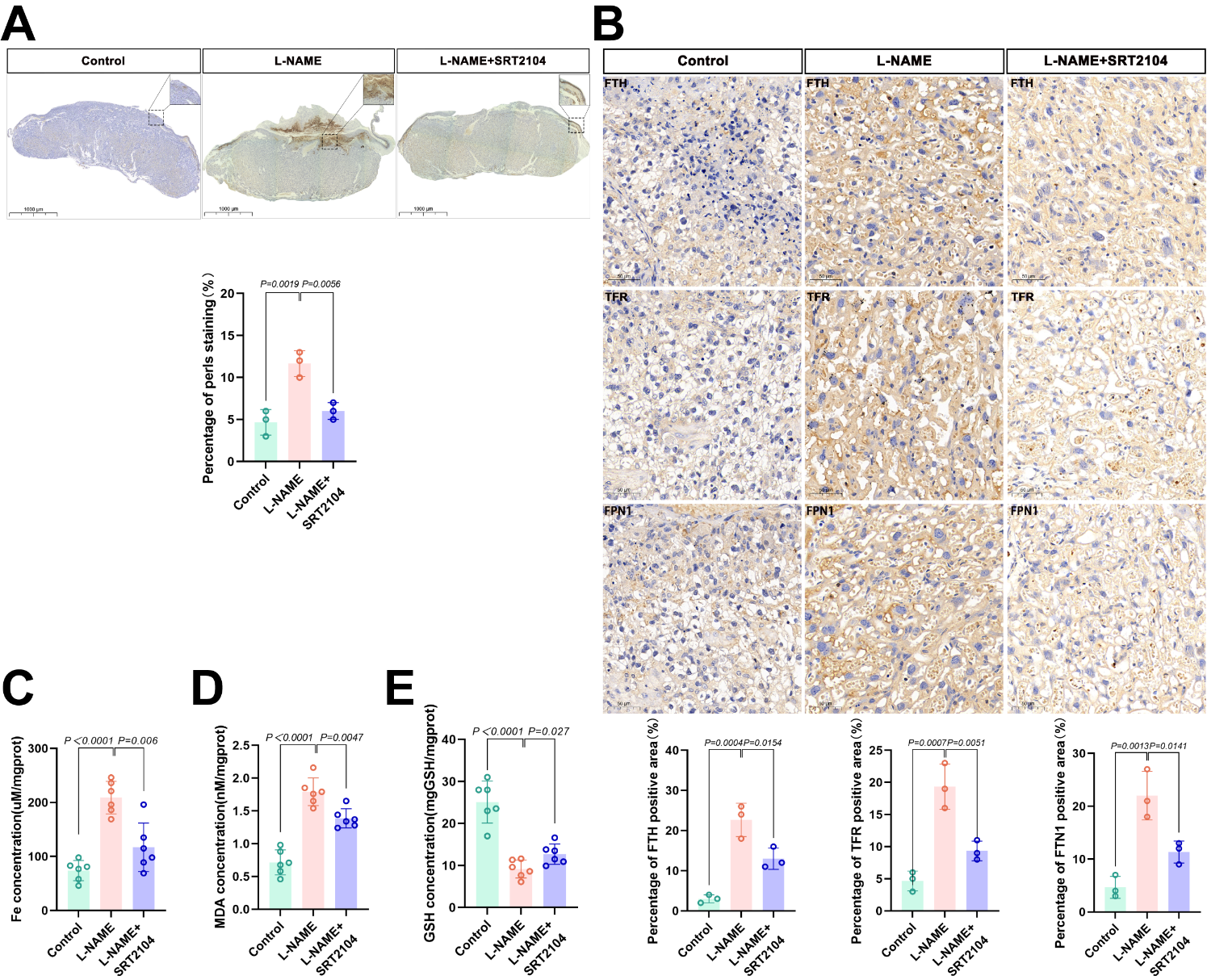


**(a)** Perls’ Prussian blue staining of placental sections at E18.5. Quantification of staining intensity per placentas; Scale bars: 1000µm. n=3. **(b)**IHC staining of FTH, TFR and FPN1 in placental sections at E18.5. Quantification of staining intensity per placentas; Scale bars: 50 μm; n=3. **(c-e)**Measurement of **(c)** iron concentration, **(d)** MDA concentration, and **(e)** GSH concentration in placental tissues at E18.5 using corresponding detection kits; n=6, one-way ANOVA and Tukey’s multiple comparison test. All data are presented as the means ± SEM.

**Table S1. Clinical characteristics of the study subjects**

|  | **Normal**（**n=30**） | **PE** (**n=30**) | **p value** |
| --- | --- | --- | --- |
| Age (years) | 29.20±2.76 | 29.80±3.10 | 0.43 |
| Gestational age (weeks) | 39.83±0.73 | 32.80±0.90 | ＜0.0001 |
| Body mass index (kg/m^2^) | 27.25±1.18 | 27.45±1.43 | 0.57 |
| Urinary protein (g/24h) | - | 3.47±1.17 | - |
| Systolic blood pressure (mmHg) | 123.6±7.08 | 157.8±8.23 | ＜0.0001 |
| Diastolic blood pressure (mmHg) | 76.30±8.19 | 111.5±7.52 | ＜0.0001 |
| Fetal birth weight (g) | 3320±346.10 | 2088±228.20 | ＜0.0001 |
| Weight of placenta (g) | 545.5±26.18 | 439.6±23.90 | ＜0.0001 |

Data are expressed as the mean ± SEM. The data were analyzed by Student’s t-test.

**Table S2. Sequences of shRNAs**

| **shRNA** | **Strand** | **Sequences (5＇→3＇)** |
| --- | --- | --- |
| SIRT1 | Sense | GGGAATCCAAAGGATAATT |
|  | Antisense | AATTATCCTTTGGATTCCC |
| NC | Sense  Antisense | TTCTCCGAACGTGTCACGT  ACGTGACACGTTCGGAGAA |

**Table S3. Sequences of siRNAs**

| **siRNA** | **Strand** | **Sequences (5＇→3＇)** |
| --- | --- | --- |
| P53 | Sense | GAUGCUACUUGACUUACGA |
|  | Antisense | UCGUAAGUCAAGUAGCAUC |
| NC | Sense | UUCUCCGAACGUGUCACGU |
|  | Antisense | ACGUGACACGUUCGGAGAA |

**Table S4. Top 5 enriched GO of differentially expressed genes**

| **GO** | | |
| --- | --- | --- |
| **Cellular component** | **Go terms** | **Genes** |
|  | Basolateral plasma membrane | TFRC/SLC41A1/SLC40A1/ABCC6/AJAP1/NDRG4/SLC6A13/LDLR/LRRC7/P2RY4/  NKD2/CLDN1/PROM2/SLC26A7/ADRA2A/MUC20/SLC6A9/NAIP/ATP12A/  BEST1/ANK3/CHRM3/EPPK1/SLC8A2/SLC16A12/CD1D |
|  | Basal part of cell membrane | TFRC/SLC41A1/SLC11A2/SLC40A1/FAP/ABCC6/AJAP1/NDRG4/SLC6A13/  LDLR/LRRC7/EDN1/P2RY4/NKD2/CLDN1/PROM2/SLC26A7/ADRA2A/  MUC20/SLC6A9/NAIP/ATP12A/BEST1/ANK3/CHRM3/EPPK1/SLC8A2/  SLC16A12/CD1D |
|  | Synaptic membrane | KCNH1/TENM2/SYT1/IL31RA/SYNE1/COL13A1/DLG4/CACNA1C/SHANK2/  RIMS2/KCTD16/P2RY4/CDH10/KCNJ3/HTR1B/GRIN2C/SYNDIG1/ADORA2A/  ADRA2A/MAGEE1/NTRK3/SLITRK3/SLC6A9/KCTD8/GRIA4/NRCAM/RAPSN/  CHRNA10/ANK3/RIMS1/CHRM3/RGS7BP/EPHA7/OPRD1/GRIN2D/NLGN1 |
|  | Postsynaptic membrane | KCNH1/TENM2/SYNE1/COL13A1/DLG4/CACNA1C/SHANK2/KCTD16/CDH10/GRIN2C/SYNDIG1/ADORA2A/ADRA2A/MAGEE1/NTRK3/SLITRK3/SLC6A9/KCTD8/  GRIA4/NRCAM/RAPSN/CHRNA10/ANK3/CHRM3/RGS7BP/EPHA7/OPRD1/  GRIN2D/NLGN1 |
|  | Collagen-containing extracellular matrix | IL7/THSD4/NTN4/NAV2/TNC/COL6A3/COL13A1/VCAN/ANGPTL4/INHBE/WNT2B/TGFB2/COL20A1/ADAMTS5/SNORC/SULF1/POSTN/LMAN1L/COL8A1/CPA3/LAMC2/PTPRZ1/VIT/GDF10/AMTN/GDF15/NCAM1/NPNT/CHADL/COL4A3/ECM2/COL17A1/LAMA3/FLG/IMPG2/LAD1/TGM4/SLPI/FRAS1 |
| **Molecular function** | Signaling receptor activator activity | IL7/IL10/TNF/SCG2/FGF5/IL6/FGF1/FLRT2/IL11/PRKCE/HMGB1/FGF2/FGF7/NRG1/CXCL8/TNFRSF11B/SPX/NGF/EDN1/ADCYAP1/SEMA5A/IL16/INHBE/STC2  /WNT2B/CGA/TGFB2/IL24/TG/VEGFA/CCL7/SEMA3E/ADM2/GPHA2/IL12B/LTB/BMP6/GDF10/GDF15/TNFSF18/GRP/EPHA7/INHBA/CXCL3/LTA/CCL5/FGF21/AREG/DAND5/STC1/NPY/GNRH1 |
|  | Receptor ligand activity | IL7/IL10/TNF/SCG2/FGF5/IL6/FGF1/FLRT2/IL11/HMGB1/FGF2/FGF7/NRG1/CXCL8/TNFRSF11B/SPX/NGF/EDN1/ADCYAP1/SEMA5A/IL16/INHBE/STC2/WNT2B/CGA/TGFB2/IL24/TG/VEGFA/CCL7/SEMA3E/ADM2/GPHA2/IL12B/LTB/BMP6/GDF10/GDF15/TNFSF18/GRP/EPHA7/INHBA/CXCL3/LTA/CCL5/FGF21/AREG/DAND5/STC1/NPY/GNRH1 |
|  | Cytokine activity | IL7/IL10/FGF5/IL6/FGF1/IL11/FGF2/FGF7/NRG1/NGF/INHBE/TGFB2/VEGFA/IL12B/BMP6/GDF10/GDF15/INHBA/FGF21/AREG |
|  | Growth factor activity | IL7/IL10/FGF5/IL6/FGF1/IL11/FGF2/FGF7/NRG1/NGF/INHBE/TGFB2/VEGFA/IL12B/BMP6/GDF10/GDF15/INHBA/FGF21/AREG |
|  | Glycosaminoglycan binding | NLRP3/FGF1/FGF2/FGF7/NAV2/CXCL8/CEMIP/TLR2/COL13A1/SLIT3/VCAN/SEMA5A/ADAMTS5/SULF1/VEGFA/POSTN/CCL7/LAMC2/VIT/SERPIND1/FGFBP1/ECM2/RSPO3/PTPRC/IMPG2 |
| **Biological process** | Iron ion homeostasis | HIF1A/HMOX1/TFRC/HMOX2/SLC11A2/NCOA4/CYBRD1/ABCB7/ATP6V1G1/SLC11A1/TTC7A/SLC40A1/FBXL5/FXN/TFR2/GLRX3/B2M/MYC/FRRS1/SLC6A9/BMP6/LCN2 |
|  | Response to iron ion | BECN1/HIF1A/HMOX1/TFRC/SNCA/ATG5/SLC11A2/CYBRD1/SLC40A1/BCL2  /FXN/TFR2/B2M/BMP6 |
|  | Cellular iron ion homeostasis | HIF1A/HMOX1/TFRC/SLC11A2/NCOA4/CYBRD1/ABCB7/ATP6V1G1/SLC11A1/  TTC7A/SLC40A1/FXN/TFR2/GLRX3/MYC/FRRS1/SLC6A9/BMP6/LCN2 |
|  | Response to oxygen levels | ATG7/PTEN/BECN1/HIF1A/HMOX1/TFRC/HMOX2/MDM2/MDM4/SLC11A2/MTOR/ARNT/TNF/TP53/PRKAA1/ATP6V1G1/OXTR/ATF4/HILPDA/BCL2/PRKCE/  GUCY1B1/TLR2/SIRT1/HK2/MYOCD/EDN1/NFE2L2/MYC/ANGPTL4/STC2/TGFB2/VEGFA/EGR1/CLDN3/CYGB/TERT/RORA/LTA/OPRD1/PTGS2/DIO3/STC1 |
|  | Epithelial cell proliferation | PTEN/HIF1A/HMOX1/IL10/ARNT/TNF/AR/SCG2/YAP1/FST/IL6/NOG/FAP/TNFAIP3/FGF1/IGFBP5/HMGB1/ZFP36/FGF2/FGF7/B2M/JUN/SNAI2/SIRT1/MAGED1/LRG1/  AGTR1/MYC/SEMA5A/BMPER/CLDN1/TGFB2/EGR3/SULF1/VEGFA/COL8A1/  HOXA5/EHF/OSR2/IL12B/TP63/LGR5/BMP6/XDH/FGFBP1/COL4A3/ESRP2/DLL4/AREG/EPPK1 |

**Table S5. Top 5 enriched KEGG pathways of differentially expressed genes**

| **KEGG** | | |
| --- | --- | --- |
| **Kegg_ID** | **Pathway** | **Genes** |
| hsa05417 | Lipid and atherosclerosis | KRAS/RELA/NLRP3/NFKB1/IRF3/ATF6/TNF/TP53/GSK3B/ATF4/TLR4/IL6/BCL2/JUN/  CXCL8/FOS/TLR2/LDLR/MMP1/MAP2K6/VLDLR/NFE2L2/HSPA2/HSPA1B/CD14/MMP3/  PIK3R3/IL12B/CYP2J2/CALML6/CXCL3/MAPK10/CCL5/LBP/NCF2 HMOX1/TP53/NCOA4/ATG5/ATG7 |
| hsa05146 | Amoebiasis | IL10/RELA/NFKB1/TNF/TLR4/IL6/CXCL8/TLR2/C8G/TGFB2/CD14/PIK3R3/IL1R2/RAB7B/  LAMC2/IL12B/SERPINB3/COL4A3/CXCL3/LAMA3/CD1D |
| hsa04140 | Autophagy - animal | ATG7/PTEN/ATG10/BECN1/HIF1A/LAMP2/ATG16L1/KRAS/AMBRA1/ATG5/ATG3/MTOR/ATG12/PRKAA1  /PRKAA2/ATG14/BCL2/HMGB1/ATG9B/IRS1/RAB39B/VAMP8/PIK3R3/RAB7B/MAPK10 |
| hsa05145 | Toxoplasmosis | IL10/RELA/NFKB1/TNF/TLR4/BCL2/SOCS1/TLR2/LDLR/MAP2K6/HSPA2/HSPA1B/TGFB2/LAMC2/  HLA-DOB/IL12B/BIRC7/LAMA3/MAPK10/IL10RA |
| hsa05167 | Kaposi sarcoma-associated herpesvirus infection | BECN1/HIF1A/KRAS/RELA/ATG3/NFKB1/MTOR/TLR3/IRF3/TP53/GSK3B/ATG14/IL6/ZFP36/FGF2/JUN/CXCL8/  FOS/RCAN1/MAP2K6/MYC/VEGFA/PIK3R3/CALML6/CXCL3/MAPK10/PTGS2 |

**Table S6. Candidate genes related to iron homeostasis identified from FerrDb and Gene Ontology databases in shSIRT1 transcriptomic analysis**

| **Candidate genes** |
| --- |
| Abcb6/Abcb7/Abcc5/Aco1/Alas2/Arhgap1/Atp13a2/Atp6ap1/Atp6v0a2/Atp6v0d1/Atp6v1a/  Atp6v1g1/Atp7a/B2m/Bola2/Bmp6/Ccdc115/Cisd1/Cltc/Cp/Cybrd1/Dnm2/Fbxl5/Flvcr1/Flvcr2/Frrs1/Fth1/  Ftmt/Fxn/Glrx3/Hamp/Hamp2/Hephl1/Heph/Hfe/Hif1a/Hmox1/Hmox2/Hsd3b2/Hsd3b3/Hsd3b6/Inhca/Ireb2/  Iscu/Lcn2/Lmtk2/Ltf/Mcoln1/Meltf/Mon1a/Mmgt1/Myc/Myo1b/Nectin1/Ncoa4/Ndfip1/  Neo1/Nos1/Nubp1/Picalm/Rep15/Sco1/Sfxn1/Slc11a1/Slc11a2/Slc25a28/Slc25a37/Slc39a8/Slc39a14/  Slc40a1/Slc48a1/Slco2b1/Snx3/Sod1/Steap1/Steap2/Steap3/Steap4/Timd2/Tfrc/Tfr2/Tmem199/Trf/Ttc7 |

**Table S7. Top 3 enriched GO and KEGG pathways of differentially expressed genes of GSE100279**

| **GO** | | |
| --- | --- | --- |
| **Cellular component** | **Go terms** | **Genes** |
|  | Extracellular membrane-bounded organelle | Prom2/Gbp3/Tmem98/Fasl/Car4 |
|  | Extracellular organelle | Prom2/Gbp3/Tmem98/Fasl/Car4 |
|  | Perinuclear endoplasmic reticulum | Cyba/Pik3r1/Gdpd5 |
| **Molecular function** | NADP-retinol dehydrogenase activity | Akr1b10/Akr1b8/Akr1b3 |
|  | Retinal dehydrogenase activity | Akr1b10/Akr1b8/Akr1b3 |
|  | Alditol:NADP+ 1-oxidoreductase activity | Akr1b10/Akr1b8/Akr1b3 |
| **Biological process** | Reactive oxygen species metabolic process | Gpx1/Sirt1/Gstp1/Sesn2/Cyba/Lcn2/Eef1a1/Gpx3/  Dcxr/Sh3pxd2b |
|  | Iron import into cell | Slc6a9/Steap2/Lcn2 |
|  | Negative regulation of Notch signaling pathway | Neurl1a/Dlk1/Chac1/Gdpd5 |
| **KEGG** | | |
| **Kegg_ID** | **Pathway** | **Genes** |
| mmu00040 | Pentose and glucuronate interconversions | Akr1b10/Akr1b8/Akr1b3/Dcxr |
| mmu00051 | Fructose and mannose metabolism | Akr1b10/Akr1b8/Akr1b3/Fbp2 |
| mmu00790 | Folate biosynthesis | Akr1b10/Akr1b8/Akr1b3 |
